# IFRD1 promotes tumor cells “low-cost” survival under glutamine starvation via inhibiting histone H1.0 nucleophagy

**DOI:** 10.1038/s41421-024-00668-x

**Published:** 2024-05-28

**Authors:** Yabin Huang, Fanzheng Meng, Taofei Zeng, Rick Francis Thorne, Lifang He, Qingrui Zha, Hairui Li, Hong Liu, Chuandong Lang, Wanxiang Xiong, Shixiang Pan, Dalong Yin, Mian Wu, Xuedan Sun, Lianxin Liu

**Affiliations:** 1 Department of Hepatobiliary Surgery, Centre for Leading Medicine and Advanced Technologies of IHM, The First Affiliated Hospital of USTC, Division of Life Sciences and Medicine, University of Science and Technology of China, Hefei, Anhui China; 2 Anhui Province Key Laboratory of Hepatopancreatobiliary Surgery, Hefei, Anhui China; 3 Anhui Provincial Clinical Research Center for Hepatobiliary Diseases, Hefei, Anhui China; 4 Translational Research Institute of People’s Hospital of Zhengzhou University and Academy of Medical Sciences, Zhengzhou University, Zhengzhou, Henan China; 5 School of Life Sciences, Division of Life Sciences and Medicine, University of Science and Technology of China, Hefei, Anhui China

**Keywords:** Cancer therapeutic resistance, Autophagy

## Abstract

Glutamine addiction represents a metabolic vulnerability of cancer cells; however, effective therapeutic targeting of the pathways involved remains to be realized. Here, we disclose the critical role of interferon-related developmental regulator 1 (IFRD1) in the adaptive survival of hepatocellular carcinoma (HCC) cells during glutamine starvation. IFRD1 is induced under glutamine starvation to inhibit autophagy by promoting the proteasomal degradation of the key autophagy regulator ATG14 in a TRIM21-dependent manner. Conversely, targeting IFRD1 in the glutamine-deprived state increases autophagy flux, triggering cancer cell exhaustive death. This effect largely results from the nucleophilic degradation of histone H1.0 and the ensuing unchecked increases in ribosome and protein biosynthesis associated with globally enhanced chromatin accessibility. Intriguingly, IFRD1 depletion in preclinical HCC models synergizes with the treatment of the glutaminase-1 selective inhibitor CB-839 to potentiate the effect of limiting glutamine. Together, our findings reveal how IFRD1 supports the adaptive survival of cancer cells under glutamine starvation, further highlighting the potential of IFRD1 as a therapeutic target in anti-cancer applications.

## Introduction

Metabolic reprogramming has been identified as one of the essential hallmarks of cancer cells^1^. In particular, glutamine metabolism provides an important source of carbon and nitrogen to sustain the growth of cancer cells^2^. The high dependence on glutamine, often called glutamine addiction, has laid the foundation for glutamine starvation therapies. Towards this notion, different approaches have been envisioned including glutamine depletion and also prevention of glutamine catabolism by inhibiting glutaminase (GLS)^3^. An alternative approach involves targeting glutamine uptake by inhibiting membrane glutamine transporters such as SLC1A5, which is upregulated in a variety of tumors^4^. Nonetheless, these strategies have currently yielded limited success. For example, the application of bacterial L-asparaginase or phenylbutyric acid to deplete systemic glutamine is only marginally effective at the early stages of tumor development^5,6^. The lack of efficacy appears to be associated with activation of the de novo glutamine synthesis pathway, and notably this has been associated with tumors developing drug resistance. Different GLS inhibitors, including Acivicin and DON have also shown promising preclinical antitumor activity, but unfortunately, these agents were discontinued after early clinical trials showed significant gastrointestinal toxicity and neurotoxicity^7,8^. Further, a GLS1 selective inhibitor (CB-839) was developed and although ineffective as monotherapy, combination therapy with CB-839 has brought survival benefits to patients with renal and colorectal cancers^3,9^. The applicability of GLS inhibitors or glutamine depletion against hepatocellular carcinoma (HCC) is still at the preclinical stage, with research showing that hepatoma cells display altered sensitivity associated with self-generated adaptive mechanisms. For example, in murine orthotopic liver tumors induced by c-MYC, GLS knockdown inhibited glutamine-derived carbon flow into the tricarboxylic acid (TCA) cycle, but this was shown to be compensated by enhanced glucose metabolism, leaving the levels of TCA cycle metabolites unaffected^10^. With respect to glutamine transport inhibition, GPNA was described as a first-generation antagonist, but it was later replaced due to low potency^11^. Given that none of the glutamine-targeting approaches have had the expected clinical impacts, it behooves us to acquire a more in-depth understanding of the molecular mechanisms that enable cancer cells to overcome glutamine starvation. Our research group has been dedicated to identifying the underlying causes of treatment resistance in glutamine deprivation therapy and has published a series of articles on this^12–14^. However, there is a lingering question. Although it is generally believed that cells would initiate autophagy in response to glutamine deprivation as a way to cope with the shortage of nitrogen and carbon sources, in reality, tumor cells do not undergo extensive autophagy despite prolonged glutamine deprivation. This suggests that tumor cells may have intrinsic mechanisms to prevent uncontrolled activation of autophagy and maintain survival in response to glutamine deprivation.

In this study, we sought to identify essential regulators that facilitate the adaptation of HCC cells to survive glutamine deprivation. Towards this goal, we employed combined transcriptomic and proteomic sequencing data to identify genes and proteins induced in HCC cells in response to limiting glutamine conditions. This approach uncovered a novel stress response mediator, namely interferon-related developmental regulator 1 (IFRD1), which was previously linked to transcriptional regulation in pathophysiological processes, including embryonic development and tissue damage repair^15–21^, although its role in cancer development and progression is poorly studied. We found that exposing HCC cells to limiting exogenous glutamine resulted in the MAFG-mediated transcriptional upregulation of IFRD1. We observed that IFRD1 mainly localizes at the endoplasmic reticulum (ER), where it inhibits autophagic flux by promoting the ubiquitination and degradation of ATG14, a key regulator of autophagy initiation. This effect is achieved by promoting the interaction between ATG14 and the E3 ubiquitin ligase TRIM21 through IFRD1. Unexpectedly, we discovered that histone H1.0, a nuclear protein, undergoes nucleophagic degradation mediated by IFRD1. As a corollary, the increased autophagy associated with *IFRD1* deletion under glutamine starvation promotes histone H1.0 degradation, thereby enhancing chromatin accessibility and expression of ribosome biosynthesis-related genes. The resulting hyperactive protein synthesis in the face of limiting exogenous glutamine sources promotes the autophagy-mediated death of HCC cells. Notably, preclinical models confirmed that combined glutamine deprivation/CB-839 treatment with IFRD1 loss caused tumor regression in vivo. Collectively, our data identify IFRD1 as a potential primary target for exploiting glutamine starvation strategies in HCC and perhaps other cancers.

## Results

### IFRD1 upregulation in response to glutamine starvation in HCC

**Fig. 1:**
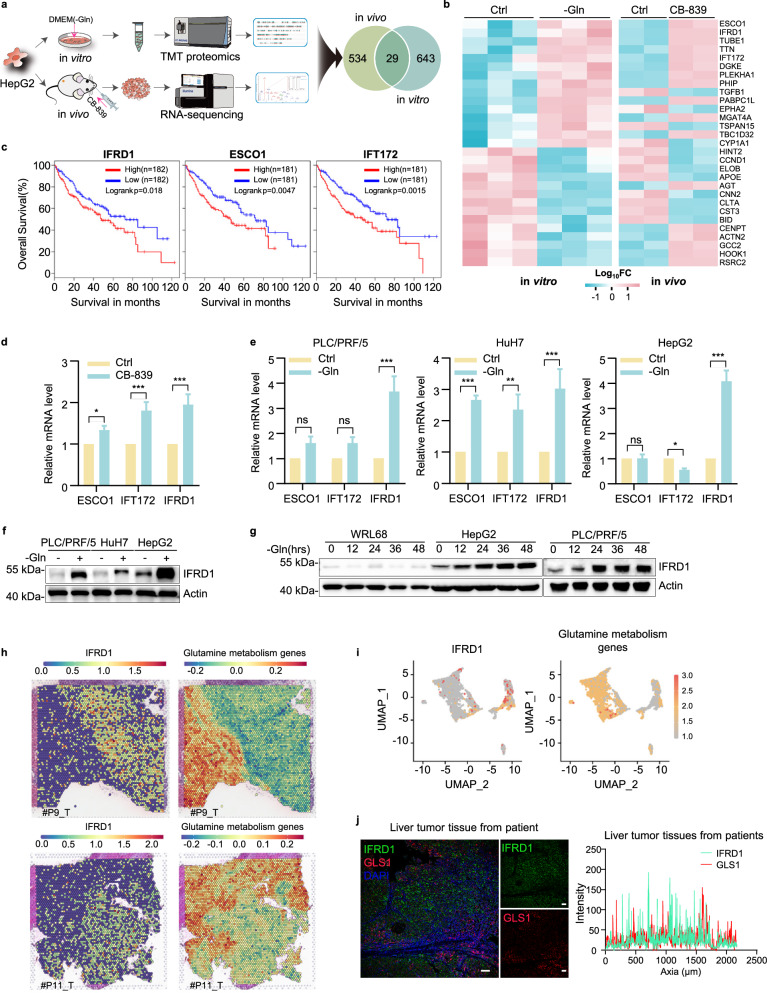
IFRD1 upregulation in response to glutamine starvation in HCC. **a** Schematic representation of in vivo and in vitro screening systems employed to identify regulators in response to glutamine starvation. For in vivo groups, significantly differentially expressed genes were screened by fold change (FC) ≥ 1.5, *P* < 0.05; for in vitro groups, significantly differentially expressed proteins were screened by FC ≥ 1.2, *P* < 0.05. **b** Heatmap showing differential expression of 29 common hits between two screening systems in **a**. The normalized FC of differentially expressed genes (DEGs) is provided in Supplementary Table S1. **c** Kaplan–Meier survival curves of overall survival in liver cancer patients from the TCGA based on IFRD1, ESCO1, and IFT172 expression. **d** Real-time quantitative PCR (RT-qPCR) analyses of *ESCO1*, *IFT172* and *IFRD1* in nude mouse bearing HepG2 tumor xenografts treated with vehicle or CB-839. **e** RT-qPCR analyses of *ESCO1*, *IFT172* and *IFRD1* in PLC/PRF/5, HuH7 and HepG2 cells cultured under normal or glutamine starvation (–Gln) conditions for 48 h. **f** Western blot analysis of expression of IFRD1 in PLC/PRF/5, HuH7, and HepG2 cells cultured with normal or glutamine-free medium for 48 h. **g** Western blot analysis of expression of IFRD1 in WRL68, HepG2, and PLC/PRF/5 cells cultured under normal or glutamine starvation conditions for the indicated hours. **h** Spatial transcriptomics data in log-normalized (logNorm) counts for IFRD1 and glutamine metabolism-related genes in tumor tissues from HCC patients. **i** UMAP plots of expression of *IFRD1* and glutamine metabolism-related genes in tumor tissues from HCC patients. **j** IFRD1 and GLS1 co-staining in tumor tissues from HCC patients. Scale bars, 100 μm. Data shown are representative of three independent experiments (**d**–**g**, **j**). Data are mean ± SD; *P < 0.05; **P < 0.01; ***P < 0.001; ns, not significant by two-tailed unpaired Student’s *t*-test or two-way ANOVA (**d**, **e**).

To identify factors involved in the adaptive survival responses of HCC cells to glutamine starvation, we hypothesized that cross-referencing data from different omics technologies and platforms would help rationalize the most promising candidate genes. Towards this, we first cultured human HepG2 cells under normal or glutamine-starved conditions and used proteomic screening to identify differentially expression proteins (Supplementary Fig. S1a). In parallel, we treated mice bearing HepG2 xenografts with the GLS inhibitor CB-839 and then took the tumor tissues for transcriptomic sequencing (Supplementary Fig. S1b). These analyses yielded 672 and 563 differentially expressed hits, respectively, and intersection analysis revealed 29 candidates in common (Fig. 1a). Further classification of the protein/gene expression changes in vitro and in vivo for consistent directional expression changes identified 19 candidates, 11 being upregulated and 8 downregulated, respectively (Fig. 1b; Supplementary Fig. S1c).

We next assessed the clinical impact of the 19 genes by performing overall survival analyses based on the TCGA-LIHC dataset. Only three genes, namely IFRD1, ESCO1, and IFT172 were able to stratify outcomes, with their high expression associated with worse overall survival (Fig. 1c; Supplementary Fig. S1d). Further validation analyses showed that among these 3 genes, IFRD1 was distinctly induced in CB-839-treated xenografts (Fig. 1d; Supplementary Figs. S1e, S2a, b), and consistently upregulated in response to glutamine deprivation in three various HCC cell lines (Fig. 1e, f) as well as CB-839-treated PLC/PRF/5 cells (Supplementary Fig. S2c, d). In addition, we further showed that glutamine starvation elevated IFRD1 levels in HCC cells in a time-dependent manner but not in normal human WRL68 hepatic cells (Fig. 1g). Interestingly, IFRD1 protein expression was also markedly stimulated under other metabolic stresses such as glucose or serum starvation in HCC cells, revealing that IFRD1 could act as a stress-responsive protein linked to cellular survival and adaptation to stress (Supplementary Fig. S3a).

Besides, we analyzed the spatial transcriptomics data on HCC^22^, and found that IFRD1 exhibited mutually exclusive spatial expression patterns with glutamine metabolism genes (Fig. 1h). Similar landscapes were observed in HCC single-cell transcriptomes^23^ (Fig. 1i). Further, immunofluorescence staining proved the differential expression patterns of IFRD1 and GLS1 in liver cancer tissue (Fig. 1j). Moreover, we were surprised to find that majority of cancer cells exhibited upregulation of IFRD1 expression upon glutamine deprivation, although there were also few cancer cells that showed no significant changes or even slight decreases in glutamine-starved conditions due to some uncertain factors (Supplementary Fig. S3b, c).

### Transcription factor MAFG induces IFRD1 expression under glutamine starvation

We first sought to explore the upstream mechanism responsible for the induction of IFRD1 under glutamine starvation conditions. Using published ATAC-seq data (ENCODE, ENCAN074ZCE), we wondered which chromatin regions were accessible in the *IFRD1* promoter region in HepG2 cells (Supplementary Fig. S4a). We interrogated potential transcription factor binding motifs in this region using the JASPAR database and further, cross-referenced these against our proteomic data of glutamine-starved HepG2 cells, which uncovered a single gene in common, namely MAFG (Supplementary Fig. S4b). Indeed, MAFG levels were consistent with the time-dependent increase in IFRD1 expression in response to glutamine starvation (Supplementary Fig. S4c), suggesting that MAFG was involved in IFRD1 expression regulation.

To test this possibility, we implemented MAFG knockdown studies. Interestingly, MAFG silencing significantly decreased IFRD1 expression in glutamine-starved HepG2 cells (Supplementary Fig. S4d, e), indicating that MAFG was required for IFRD1 upregulation. To strengthen these findings, we designed luciferase reporter vectors and found that ectopic expression of MAFG enhanced the transcriptional activity of the *IFRD1* promoter, whereas deletion of the MAFG motif completely abolished the increased activity (Supplementary Fig. S4f, g). Moreover, as anticipated, the transcriptional activity of the wild-type (WT) *IFRD1* promoter construct was stimulated following glutamine starvation, but no changes occurred in cells where expression of MAFG was restrained by knockdown with shRNA (Supplementary Fig. S4h). Furthermore, using chromatin immunoprecipitation (ChIP) assays, we confirmed that endogenous MAFG directly binds to the *IFRD1* promoter (Supplementary Fig. S4i). Thus, MAFG directly transactivates IFRD1 during glutamine starvation.

### *IFRD1* deletion enhances autophagy flux under glutamine starvation

**Fig. 2:**
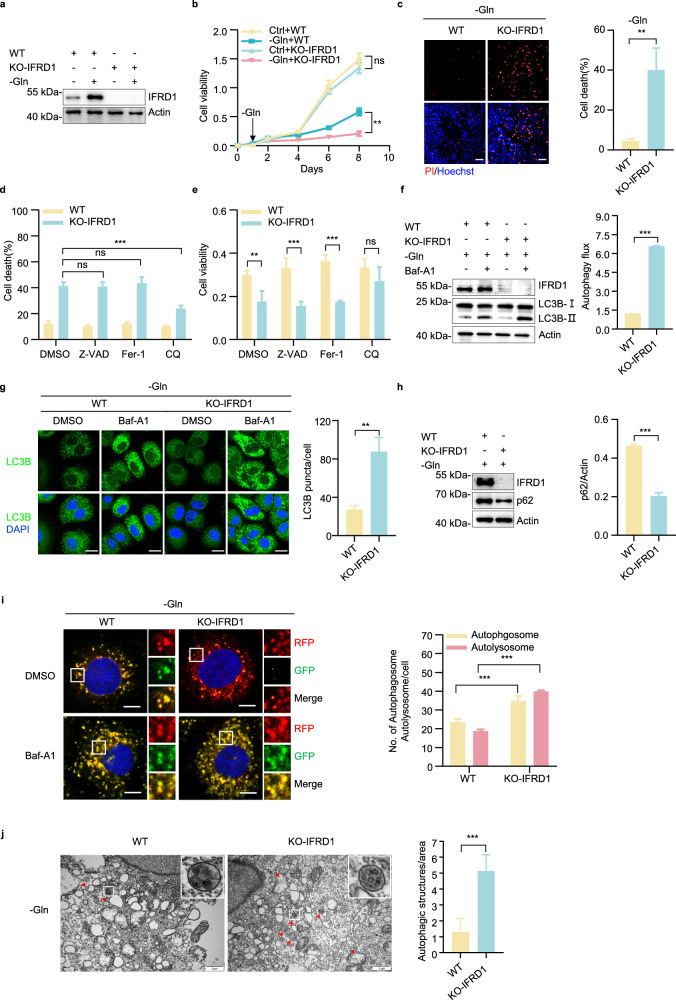
*IFRD1* deletion enhances autophagy flux and promotes cell death under glutamine starvation. **a** Western blot analysis of IFRD1 expression in WT and KO-IFRD1 PLC/PRF/5 cells cultured under normal or glutamine starvation conditions for 48 h. **b** Cell viability of WT and KO-IFRD1 PLC/PRF/5 cells under normal or glutamine starvation conditions. Arrow indicates the time point that glutamine starvation started. **c** Fluorescence microscopy images of WT and KO-IFRD1 PLC/PRF/5 cells cultured with glutamine-deprived medium for 48 h followed by double staining with propidium iodide (PI) and Hoechst 33342 (left). PI and Hoechst 33342 stained cells were counted, and the percentage of PI-positive cells was then quantified. Scale bar, 50 μm. **d** Quantification of percentage of PI-positive cells in WT and KO-IFRD1 PLC/PRF/5 cells under glutamine starvation conditions for 48 h with the addition of DMSO, Z-VAD (20 μM), Fer-1 (1 μM), and CQ (10 μM) for the last 24 h, respectively. **e** Cell viability of WT and KO-IFRD1 PLC/PRF/5 cells under glutamine starvation conditions with the addition of DMSO, Z-VAD (20 μM), Fer-1 (1 μM), and CQ (10 μM) for 5 days, respectively. **f** Representative immunoblots of LC3B and actin (loading control) in WT and KO-IFRD1 PLC/PRF/5 cells under glutamine starvation conditions for 36 h with or without the addition of Baf-A1 (400 nM) for the last 2 h (left). The ratio of (LC3B-II (+Baf-A1)/actin)/(LC3B-II (–Baf-A1)/actin) indicates autophagy flux (right). **g** Representative images of LC3B immunostaining in WT and KO-IFRD1 PLC/PRF/5 cells under glutamine starvation conditions for 36 h with the addition of DMSO or Baf-A1 (400 nM) for the last 2 h (left). DAPI staining indicates nucleus. Percentage of LC3B puncta per cell in WT and KO-IFRD1 PLC/PRF/5 cells (glutamine-starved for 36 h) with Baf-A1 was quantified (*n* = 5) (right). Scale bars, 20 μm. **h** Representative immunoblots of the indicated proteins in WT and KO-IFRD1 PLC/PRF/5 cells under glutamine starvation conditions for 36 h (left). Ratios of p62/actin were quantified (right). **i** Representative confocal images of WT and KO-IFRD1 PLC/PRF/5 cells expressing mRFP-GFP-LC3 under glutamine starvation conditions for 36 h with the addition of DMSO or Baf-A1 (400 nM) for the last 2 h (left). Insets: magnified views of the regions in the white boxes. Quantification of LC3B puncta representing autophagosomes (yellow) and autolysosomes (red) in cells (*n* = 6) (right). Scale bars, 10 μm. **j** Representative electronic micrographs of autophagosomes or autolysosomes of WT and KO-IFRD1 PLC/PRF/5 cells under glutamine starvation conditions for 36 h. Red arrows indicate autophagic structures. Insets: magnified views of the regions in the white boxes (left). The number of autophagic structures per area was quantified (*n* = 6) (right). Scale bars, 1 µm. Data shown are representative of three independent experiments. Data are mean ± SD. **P < 0.01, ***P < 0.001; ns, not significant by two-tailed unpaired Student’s *t* test or two-way ANOVA.

It was important to ensure that the induction of IFRD1 following glutamine withdrawal had meaningful phenotypic effects on HCC cells. Towards this, we utilized CRISPR-Cas9 technology to derive PLC/PRF/5 cells lacking IFRD1 expression (KO-IFRD1) (Fig. 2a). Intriguingly, deletion of *IFRD1* resulted in decreased cell viability and dramatically increased cell death, suggesting that IFRD1 is essential for the adaptive survival of HCC cells under glutamine starvation, while having no impact on cell growth under normal conditions (Fig. 2b, c; Supplementary Fig. S5a, b). A similar effect was observed under the treatment of CB-839 (Supplementary Fig. S6a, b). Based on these findings, we focused our efforts on investigating the role of IFRD1 in promoting HCC cell survival under glutamine starvation conditions.

We next investigated the underlying reasons why knockout of IFRD1 led to increased cell death following glutamine withdrawal. The major classical cell death pathways include apoptosis, ferroptosis, and autophagic cell death, all of which can be distinguished using specific inhibitors^24^. Interestingly, cell death in IFRD1 knockout cells induced by glutamine deprivation was significantly attenuated by treating cells with chloroquine (CQ; autophagy inhibitor) but not by treatment with Z-VAD (OMe)-FMK (Z-VAD; apoptosis inhibitor) or ferrostatin-1 (Fer-1; ferroptosis inhibitor) (Fig. 2d, e). Moreover, expression of the typical markers of apoptosis (caspase-3) and ferroptosis (GPX4) was unaffected in *IFRD1*-deleted cells under glutamine starvation (Supplementary Fig. S7a, b). Thus, these findings indicate that IFRD1 plays a protective role against autophagic cell death induced by glutamine starvation.

To verify that IFRD1 modulates autophagy under glutamine starvation, we first compared the expression of the autophagosome marker LC3B-II in WT vs IFRD1 knockout PLC/PRF/5 cells without and with the addition of the autophagy inhibitor Bafilomycin A1 (Baf-A1). As expected, Baf-A1 treatment in concert with glutamine starvation increased the proportion of LC3B-II in WT cells while the accumulation of LC3B-II was exacerbated in IFRD1 knockout cells (Fig. 2f), suggesting an elevation in autophagy flux, which was also observed in CB-839-treated KO-IFRD1 PLC/PRF/5 cells (Supplementary Fig. S6c). Immunofluorescence also revealed the expected increase in LC3B staining intensity in response to Baf-A1 (Fig. 2g). Moreover, the level of p62, which accumulates in cells with impaired autophagy, was reduced in glutamine-starved IFRD1 knockout cells (Fig. 2h). Further assessment of autophagy was undertaken using the tandem mRFP-GFP-LC3B reporter which distinguishes between autophagosomes (GFP-positive/RFP-positive, yellow merged puncta) and their subsequent maturation into autolysosomes (GFP-negative/RFP-positive, red merged puncta). We found that *IFRD1* deletion in PLC/PRF/5 cells promoted the generation of autophagosomes and autolysosomes under glutamine starvation conditions (Fig. 2i). Consistently, utilizing transmission electron microscopy, we observed a significant increase in autophagic vacuoles in IFRD1 knockout cells (Fig. 2j). Collectively, these findings establish that IFRD1 functions to inhibit autophagy flux and autophagic cell death in response to glutamine starvation.

### IFRD1 inhibits autophagy through downregulation of ATG14

**Fig. 3:**
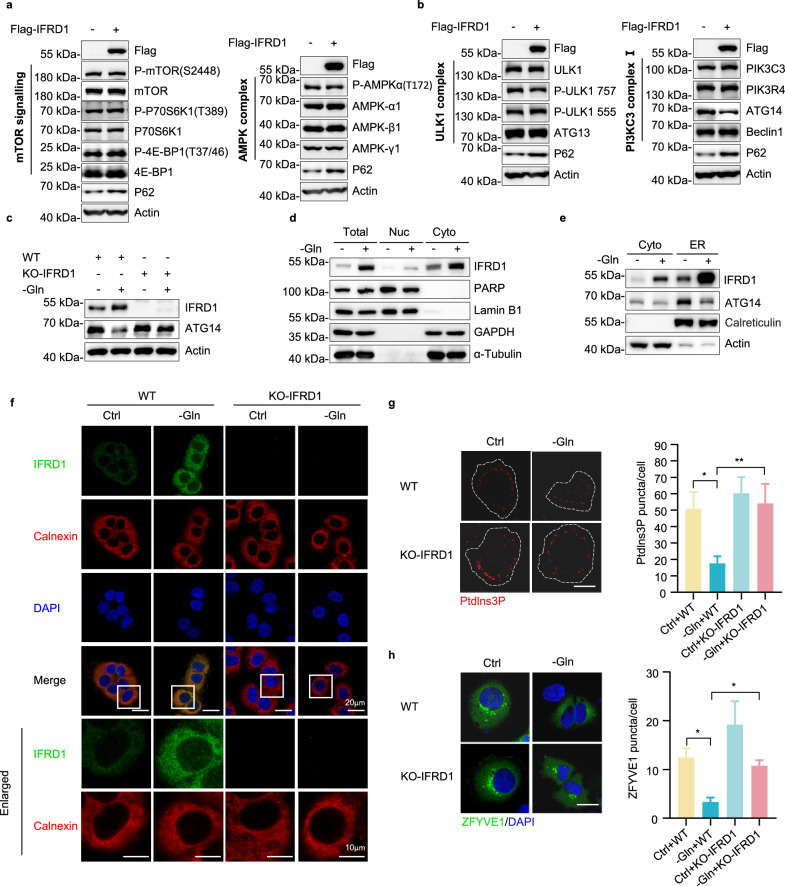
IFRD1 inhibits autophagy through downregulation of ATG14. **a** Representative immunoblots of the indicated autophagy-related regulatory factors in HepG2 cells expressing Flag or Flag-IFRD1. **b** Representative immunoblots of ULK1 complex and PI3KC3 complex I in HepG2 cells expressing Flag or Flag-IFRD1. **c** Representative immunoblots of ATG14 in WT and KO-IFRD1 PLC/PRF/5 cells cultured with normal or glutamine-free medium for 48 h. **d** Representative immunoblots of IFRD1 in the cytoplasm (Cyto)- and nucleus (Nuc)-enriched fractions extracted from normal or glutamine-starved HepG2 cells. PARP and lamin B1 are markers of nucleus; GAPDH and α-tubulin are cytoplasm markers. **e** Representative immunoblots of IFRD1 and ATG14 in the cytoplasm (Cyto)- and ER-enriched fractions extracted from normal or glutamine-starved HepG2 cells. Calreticulin is ER marker; actin is cytoplasm marker. **f** Representative confocal images of IFRD1 in WT and KO-IFRD1 PLC/PRF/5 cultured with normal or glutamine-deprived medium for 48 h. Calnexin staining indicates ER; DAPI staining indicates nucleus. Insets: magnified views of the regions in the white boxes. **g**, **h** Representative confocal images of Ptdlns3P and ZFYVE1-GFP puncta in WT and KO-IFRD1 PLC/PRF/5 cells cultured with normal or glutamine-free medium for 48 h (left). The numbers of Ptdlns3P and ZFYVE1-GFP puncta were quantified per cell (*n* = 5) (right). Scale bars, 20 µm. Data shown are representative of three independent experiments. Data are mean ± SD; *P < 0.05; **P < 0.01 by two-way ANOVA (**g**, **h**).

We next investigated the mechanism underlying the role of IFRD1 in suppressing autophagy. Previous studies have shown that mTOR and AMPK act as central regulators in autophagy induction^25,26^. Nevertheless, systematic dissection of the phosphorylation of key mTOR signaling components or AMPK complex components showed that IFRD1 expression did not influence mTOR or AMPK activity (Fig. 3a). However, when examining regulatory elements of autophagy initiation, which involves genes associated with the ULK1 complex and PI3KC3 complex I, respectively^27^, we found that ATG14, the crucial member of the PI3KC3 complex I, was decreased in HepG2 cells overexpressing IFRD1 (Fig. 3b). Strikingly, ATG14 levels were reduced in glutamine-starved PLC/PRF/5 cells while its levels remained unaffected in IFRD1 knockout cells (Fig. 3c). Together, these findings suggest that IFRD1 influences autophagy through downregulation of ATG14.

ATG14 is responsible for targeting the PI3KC3 complex to the ER, an essential event in autophagosome formation^28^. Thus, we explored how glutamine starvation affects the localization of IFRD1 in HCC cells. Using cell fractionation, we observed that IFRD1 was predominantly detected in cytoplasmic fractions, which increased upon glutamine withdrawal, while only a minor amount in nuclear fractions (Fig. 3d). Further fractionation to distinguish between cytoplasmic and ER components revealed a substantial pool of ER-associated IFRD1 protein which increased in cells subjected to glutamine starvation. In contrast, glutamine deprivation was associated with decreased localization of ATG14 to the ER (Fig. 3e; Supplementary Fig. S9a). Consistently, confocal microscopy analyses revealed significant elevations in cytoplasmic IFRD1 staining, which substantially overlapped with the ER marker calnexin staining (Fig. 3f), indicating that IFRD1 localizes to the ER.

As a corollary to these experiments, we predicted that IFRD1 would impact the activity of the PI3KC3 complex I. Towards this, we monitored the effects of IFRD1 on phagophore (early-stage autophagosome) formation using immunofluorescence staining against the phagophore markers, PtdIns3P and ZFYVE1. This analysis revealed that glutamine starvation led to reduced numbers of phagophores as indicated by the decreased numbers of PtdIns3P and ZFYVE1-GFP puncta, which was rescued in IFRD1 knockout cells (Fig. 3g, h). These observations confirmed that IFRD1 inhibits autophagy through inhibition of PI3KC3 complex I activity, although the precise mechanism whereby IFRD1 influences ATG14 levels remains to be determined.

### IFRD1 facilitates ATG14 protein degradation via TRIM21-mediated ubiquitination in response to glutamine starvation

**Fig. 4:**
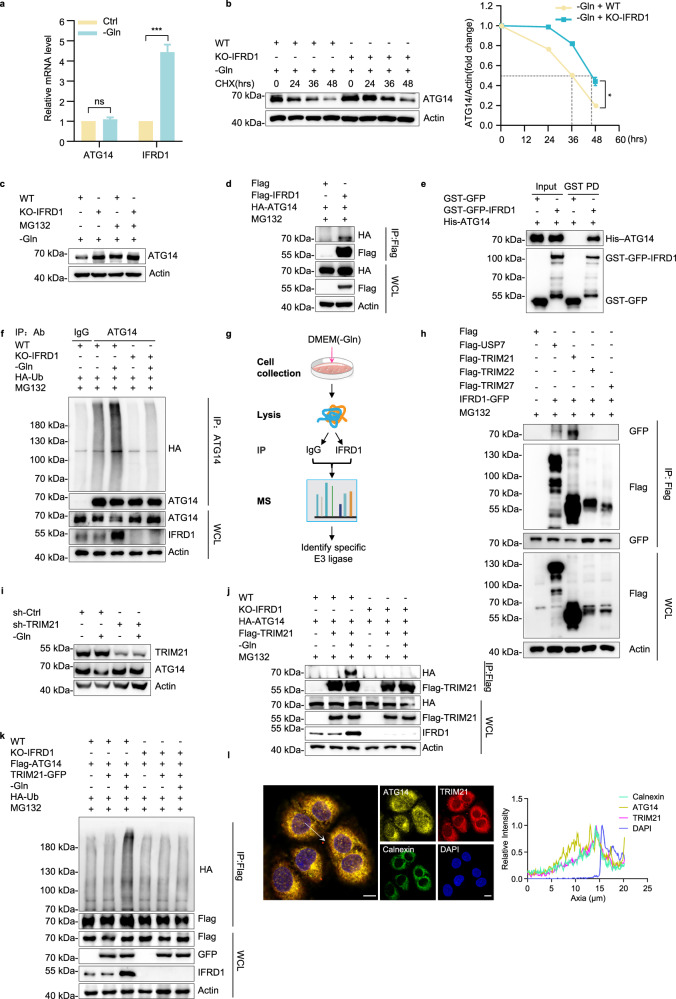
IFRD1 facilitates ATG14 protein degradation via TRIM21-mediated ubiquitination in response to glutamine deprivation. **a** RT-qPCR analyses of *IFRD1* and *ATG14* in HepG2 cells cultured with normal or glutamine-free medium for 48 h. **b** Representative immunoblots of ATG14 in WT and KO-IFRD1 PLC/PRF/5 cells under normal or glutamine starvation conditions for 48 h with the addition of CHX (50 µg/mL) for the indicated hours (left). The half-life of ATG14 was quantified by ratio of ATG14/actin (right). **c** Western blot analysis of ATG14 expression in WT and KO-IFRD1 PLC/PRF/5 cultured under glutamine starvation conditions for 36 h with the addition of MG132 for the last 10 h. **d** Co-IP analysis of the interaction between IFRD1 and ATG14 in HepG2 cells. **e** The direct interaction of IFRD1 and ATG14 was assayed by GST pull-down (GST PD) assays followed by immunoblot analysis. **f** Ubiquitination level of ATG14 in WT and KO-IFRD1 PLC/PRF/5 cells cultured with normal or glutamine-deprived medium for 36 h. **g** IP-MS strategy for identification of the potential E3 ligases that mediate the IFRD1-induced degradation of ATG14. MS results are shown in Supplementary Table S4. **h** Co-IP analysis of the interaction between IFRD1 and potential E3 ligases in HepG2 cells. **i** Representative immunoblots of ATG14 in sh-Ctrl or sh-*TRIM21* treated HepG2 cells cultured with normal or glutamine-free medium for 36 h. **j** IP analyses of the interaction of TRIM21 and ATG14 in WT and KO-IFRD1 PLC/PRF/5 cells cultured with normal or glutamine-deprived medium for 36 h. **k** IP analyses of TRIM21-mediated ubiquitination of ATG14 in WT and KO-IFRD1 PLC/PRF/5 cells cultured with normal or glutamine-free medium for 36 h. **l** Representative immunofluorescence images of ATG14, TRIM21, and Calnexin in PLC/PRF/5 cells cultured under glutamine starvation conditions for 36 h. The fluorescence intensities of ATG14 and TRIM21 signals were measured at the location of the white arrow, in the direction from arrow tail to tip. Scale bars, 10 µm. Data shown are representative of three independent experiments (**a**–**f**, **h**–**l**). Data are mean ± SD; ***P < 0.001; ns, not significant by two-way ANOVA (**a**, **b**).

We next determined whether IFRD1 influences ATG14 levels via transcriptional, translational, or post-translational mechanisms. Intriguingly, there were no changes in ATG14 mRNA levels upon glutamine starvation (Fig. 4a), excluding transcriptional regulation of ATG14. However, examination of the stability of the ATG14 protein using cycloheximide (CHX) to inhibit protein synthesis revealed that the half-life of the ATG14 protein was prolonged in IFRD1 knockout PLC/PRF/5 cells under glutamine starvation (Fig. 4b). Further, to determine whether ATG14 degradation occurs through proteasome-dependent mechanisms, we treated cells with proteasome inhibitor MG132. Indeed, MG132 treatment prevented the degradation of ATG14 under glutamine starvation conditions (Fig. 4c), suggesting that IFRD1 promotes ATG14 protein turnover via the ubiquitin-proteasome pathway.

To test whether IFRD1 directly binds to ATG14, we performed co-immunoprecipitation (co-IP) and GST pull-down assays. Notably, co-IP assays revealed robust reciprocal interaction between IFRD1 and ATG14 proteins ectopically expressed in HepG2 cells (Fig. 4d; Supplementary Fig. S8a). Moreover, GST pull-down assays confirmed direct interaction between IFRD1 and ATG14 (Fig. 4e). Parallel assays to assess the ubiquitination status of ATG14 showed that polyubiquitination of ATG14 was increased in cells overexpressing IFRD1 (Supplementary Fig. S8b); similarly, glutamine starvation induced the marked increase in polyubiquitination of endogenous ATG14 in WT but not IFRD1 knockout cells (Fig. 4f). Next, truncated versions of ATG14 were used to map the specific domains interacting with IFRD1. As shown in Supplementary Fig. S8c, d, the coiled-coil domain of ATG14 protein is primarily responsible for their interaction. Collectively, these experiments confirmed the relationship between glutamine deprivation, IFRD1 expression, and the proteasome-mediated turnover of ATG14.

To ascertain the enzymes responsible for catalyzing ATG14 ubiquitination in concert with IFRD1, we performed immunoprecipitation-coupled mass spectrometry (IP-MS) against IFRD1. We identified 4 potential E3 ligases/deubiquitinases (USP7, TRIM21, TRIM22 and TRIM27) that have not been reported to bind IFRD1 (Fig. 4g; Supplementary Table S3). Co-IP between ectopically expressed candidates and IFRD1 in HepG2 cells showed that only TRIM21 had robust interaction with IFRD1 (Fig. 4h), thus prompting us to choose TRIM21 as the primary E3 ligase. Interestingly, further fractionation to distinguish between cytoplasmic and ER components revealed a substantial pool of ER-associated TRIM21 protein, which increased in cells subjected to glutamine starvation (Supplementary Fig. S9b). Furthermore, overexpression of TRIM21 led to a remarkable decline in endogenous ATG14 levels (Supplementary Fig. S10a). Moreover, the reduction in ATG14 levels in response to glutamine deprivation was ablated following the knockdown of TRIM21 (Fig. 4i).

Our initial hypothesis was that IFRD1 acts as a regulator to facilitate engagement between ATG14 and an unidentified E3 ligase now revealed as TRIM21. Providing further evidence for this postulate, we found that the expression of IFRD1 was essential for the interaction between TRIM21 and ATG14 (Supplementary Fig. S10b). In addition, the increase in polyubiquitination of ATG14 by TRIM21 required the presence of IFRD1 (Supplementary Fig. S10c). Conversely, the increased TRIM21–ATG14 interaction and TRIM21-mediated polyubiquitination of ATG14 observed under glutamine starvation conditions were both markedly weakened in IFRD1 knockout cells (Fig. 4j, k). Moreover, we found that the ubiquitination site on ATG14 does not overlap with the interaction domain of ATG14 with IFRD1 (Supplementary Fig. S8e). Co-localization of ATG14 and TRIM21 on ER was also observed under glutamine starvation (Fig. 4l). Finally, two-hybrid assays confirmed that reporter activity measuring the interaction between pACT-TRIM21 and pBIND-ATG14 was significantly increased after ectopic expression of IFRD1 (Supplementary Fig. S9c).

Collectively, these data indicate that the increase in IFRD1 levels induced by glutamine starvation restrains autophagy, and IFRD1 acts directly as a scaffold that recruits TRIM21 to interact with ATG14, resulting in the proteasomal degradation of ATG14.

### *IFRD1* deletion promotes nucleophagic degradation of histone H1.0 under glutamine starvation

**Fig. 5:**
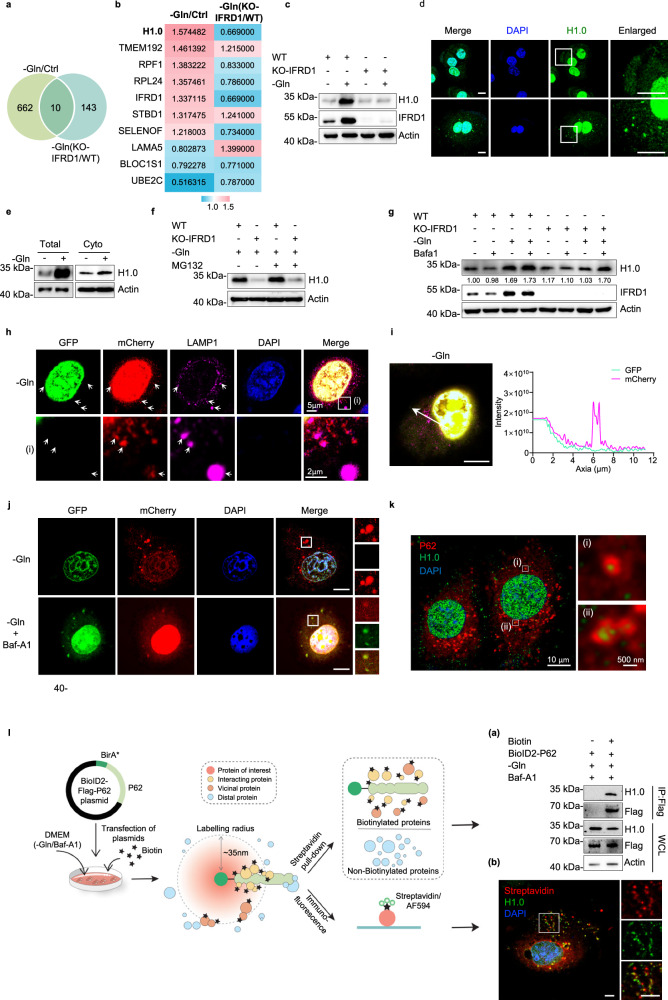
*IFRD1* deletion promotes nucleophagic degradation of H1.0 under glutamine starvation. **a** Overlap of glutamine-starved vs normal HepG2 proteome data sets (FC ≥ 1.2, *P* < 0.05) and glutamine-starved IFRD1 knockout vs WT HepG2 proteome data sets (FC ≥ 1.2, *P* < 0.05). The FC of DEGs is provided in Supplementary Table S5. **b** Heatmap showing differential expression of 10 common hits between two screening systems in **a**. **c** Representative immunoblots of H1.0 in WT and KO-IFRD1 PLC/PRF/5 cells under normal or glutamine starvation conditions for 36 h. **d** Representative confocal images of H1.0 in WT and KO-IFRD1 PLC/PRF/5 cells cultured with normal or glutamine-free medium for 36 h. Insets: magnified views of the regions in the white boxes. Scale bars, 10 µm. **e** Representative immunoblots of H1.0 in the total and cytoplasm (Cyto)-enriched fractions extracted from normal or glutamine-starved PLC/PRF/5 cells. **f** Western blot analysis of H1.0 expression in WT and KO-IFRD1 PLC/PRF/5 cultured under glutamine starvation conditions for 36 h with the addition of MG132 for the last 10 h. **g** Representative immunoblots of H1.0 in WT and KO-IFRD1 PLC/PRF/5 cells under normal or glutamine starvation conditions for 36 h with or without the addition of Baf-A1 (400 nM) for the last 2 h. **h** Representative confocal images of PLC/PRF/5 cells transfected with mCherry-GFP-H1.0 under glutamine starvation conditions for 36 h with the addition of Baf-A1 (400 nM) for the last 2 h. **i** The relative intensities of the mCherry and GFP signals of a typical cell as in **h**. The fluorescence intensities of GFP and mCherry signals were measured at the location of the white arrow, in the direction from arrow tail to tip. Scale bars, 10 µm. **j** Representative confocal images of PLC/PRF/5 cells transfected with mCherry-GFP-H1.0 under glutamine starvation conditions for 36 h with or without the addition of Baf-A1 (400 nM) for the last 2 h. Insets: magnified views of the regions in the white boxes. Scale bar, 10 µm. **k** Representative images of PLC/PRF/5 cells cultured under glutamine starvation conditions for 36 h with the addition of Baf-A1 (400 nM) for the last 2 h. **l** Scheme of proximal biotinylation and Flag-BirA* tagged p62. PLC/PRF/5 cells transfected with BioID2-p62 plasmid were cultured under glutamine starvation conditions for 36 h and incubated with biotin (4 nM) for the last 24 h with the addition of Baf-A1 (400 nM) for 2 h. Cells were collected and subjected to pull-down with streptavidin-conjugated beads (**a**) and immunofluorescence staining (**b**). Scale bars, 5 µm. Data shown are representative of three independent experiments (**c**–**l**). In **h**, **j**, **k**, **l** (**b**) white boxes: magnified views of the regions, and the corresponding scales are shown in the figure. In **h**, the arrows indicate cytoplasmic H1.0 puncta with strong mCherry signals and fading GFP signals.

As noted above, autophagy functions to promote cell survival but can also trigger cell death, although the precise circumstances behind this cell fate switch are not completely understood. Here, we found that glutamine deprivation stress, in concert with excessive autophagy associated with IFRD1 loss, was sufficient to trigger cell death. We exploited this to better understand how autophagy in this circumstance promotes cell death. We again turned to our proteomic screening data to compare the effects of glutamine starvation along with IFRD1 knockout on HCC cells. We proposed that proteins involved in overcoming glutamine starvation-induced cell death would be upregulated in WT cells but likely downregulated in IFRD1 knockout cells. By comparing the list of 672 differentially expressed proteins in WT cells under glutamine deprivation versus control conditions (–Gln/Ctrl) against the 153 differentially expressed proteins in glutamine-starved IFRD1 knockout vs WT cells (–Gln (KO-IFRD1/WT)), we derived a shortlist of 10 candidates including IFRD1 (Fig. 5a, b). Cursory analysis showed that changes in the expression of 4 candidate proteins (H1.0, RPF1, RPL24, and SELENOF) were consistent with that of IFRD1. Among them, histone H1.0 was most significantly upregulated under glutamine starvation, while being most reduced when *IFRD1* was deleted. Further, we verified that H1.0 protein levels were elevated in response to glutamine deprivation but reduced in IFRD1 knockout cells (Fig. 5c). Interestingly, we observed H1.0 puncta outside of nucleus upon glutamine starvation (Fig. 5d, e).

Next, we explored the mechanistic model of IFRD1 regulation on H1.0 expression. IFRD1 knockout-mediated H1.0 reduction was unaffected by treatment of MG132, thus excluding proteasome-dependent degradation of H1.0 (Fig. 5f). Moreover, inhibiting autophagy with Baf-A1 in combination with glutamine starvation increased H1.0 levels in IFRD1 knockout but not WT cells (Fig. 5g), which revealed that, possibly, IFRD1 loss decreases H1.0 expression in an autophagy-dependent manner. We then used an mCherry-GFP-H1.0 tandem-tag construct to investigate the autophagic trafficking of H1.0. Due to the sensitivity of GFP to low pH, mCherry-only signals of the tandem-tagged protein represent localization within acidic autolysosomes and lysosomes. Confocal imaging of mCherry-GFP-H1.0 expressed in glutamine-starved PLC/PRF/5 cells showed predominantly nuclear localization but cytoplasmic red-only H1.0 puncta that co-localized with LAMP1 (Fig. 5h, i). Blockage of the fusion between autophagosomes and lysosomes with Baf-A1 prevented GFP quenching from the mCherry-GFP-H1.0 protein in glutamine-deprived PLC/PRF/5 cells and led to the retention of its merged yellow signals in the cytoplasm (Fig. 5j), which indicates that cytoplasmic H1.0 is targeted by autophagic degradation.

As binding to autophagic receptor is essential for degradation of autophagy substrates, we investigated potential interactions between H1.0 and autophagic receptor. Using high-resolution microscopic imaging, H1.0 was found to reside in autophagosomes as indicated by co-localization with classic autophagic receptor p62 (Fig. 5k). Further, we applied a BioID2 method to visualize the potential interactions between H1.0 and p62 in glutamine-starved PLC/PRF/5 cells. The result indicates that p62 binds to H1.0, and they exhibit co-localization (Fig. 5l). Together, these data confirmed that IFRD1 inhibits the nucleophagic degradation of H1.0 under glutamine starvation.

### *IFRD1* deletion promotes protein synthesis via enhancing chromatin accessibility under glutamine starvation

**Fig. 6:**
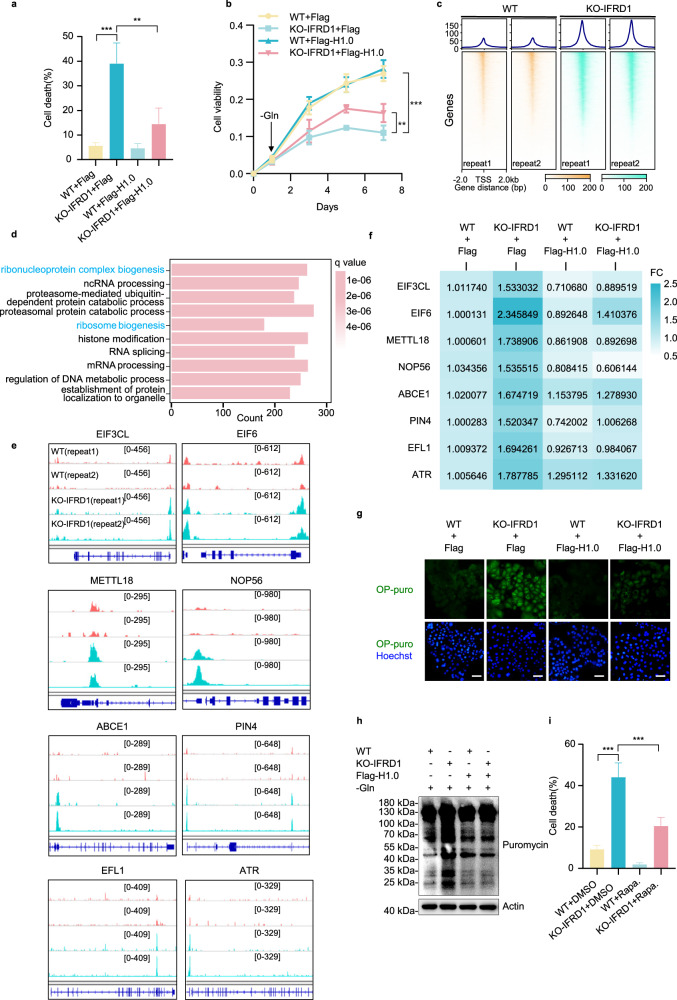
*IFRD1* deletion promotes protein synthesis via enhancing chromatin accessibility under glutamine starvation. **a** Quantification of the percentage of PI-positive cells in WT and KO-IFRD1 PLC/PRF/5 cells expressing Flag or Flag-H1.0 cultured under glutamine starvation conditions for 48 h. **b** Cell viability of WT and KO-IFRD1 PLC/PRF/5 cells expressing Flag or Flag-H1.0 cultured under glutamine starvation conditions. Arrow indicates the time point that glutamine starvation started. **c** Comparative genome-wide chromatin accessibility signals at TSSs (TSS ± 2 kb) based on ATAC-seq in WT and KO-IFRD1 PLC/PRF/5 cells. **d** The top 10 significant GO-BP terms from promoter region accessibility analysis of upregulated genes between WT and KO-IFRD1 cells cultured under glutamine starvation conditions. **e** Genome browser traces of ATAC-seq peaks at ribosome biogenesis-related gene loci. **f** RT-qPCR analyses of ribosome biogenesis-related genes in WT and KO-IFRD1 PLC/PRF/5 cells expressing Flag or Flag-H1.0 cultured under glutamine starvation conditions for 36 h. **g** Representative immunostaining images of nascent protein-OP-puro (puromycin) conjugates in WT and KO-IFRD1 PLC/PRF/5 cells expressing Flag or Flag-H1.0 cultured under glutamine starvation conditions for 36 h. Scale bars, 100 µm. **h** Protein synthesis (assessed by puromycin pulse-chase) in WT and KO-IFRD1 PLC/PRF/5 cells expressing Flag or Flag-H1.0 cultured under glutamine starvation conditions for 36 h. **i** Quantification of percentage of PI-positive cells in WT and KO-IFRD1 PLC/PRF/5 cells cultured under glutamine starvation conditions for 36 h with the addition of DMSO or rapamycin (5 μM) for the last 24 h. Data shown are representative of three independent experiments (**a**, **b**, **f**–**i**). Data are mean ± SD; **P < 0.01; ***P < 0.001 by two-way ANOVA (**a**, **b**, **i**).

Notably, overexpression of H1.0 largely rescued cell death in IFRD1 knockout cells subjected to glutamine deprivation (Fig. 6a; Supplementary Fig. S11a) and enhanced the growth of IFRD1 knockout cells after glutamine withdrawal (Fig. 6b; Supplementary Fig. S11b). We next sought to uncover why changes in H1.0 expression were sufficient to permit survival under glutamine starvation conditions.

Histones are the primary structural proteins of chromosomal organization, and H1.0 is involved in heterochromatin formation, epigenetic regulation, gene transcription, and chromosome remodeling^29,30^. Hence, we speculated that in the absence of glutamine, increased H1.0 expression and the resulting chromatin compaction places cells in a “silent” state, providing a “low-cost” mechanism to prevent cell catastrophe. Thus, under glutamine starvation, downregulation of H1.0 disrupts this homeostasis, leading to autophagic cell death. Clear evidence was obtained using ATAC-seq, which showed that genome-wide chromatin accessibility in gene promoter regions was enhanced in IFRD1 knockout compared to WT PLC/PRF/5 cells (Fig. 6c; Supplementary Fig. S12a, b). Moreover, we used confocal microscopy to examine heterochromatin by staining against H3K9me3^31^. Interestingly, *IFRD1* deletion resulted in reduced heterochromatin at the nuclear periphery during glutamine starvation, whereas ectopic expression of H1.0 prominently reversed this reduction (Supplementary Fig. S12c). Together, these data demonstrate that IFRD1 enhances chromatin accessibility under glutamine-limiting conditions by promoting H1.0 expression.

Next, we performed enrichment analyses based on the differential promoter peaks determined from the ATAC-seq data. Interestingly, general biological processes including ribosome biosynthesis-related pathways were significantly enriched in IFRD1 knockout PLC/PRF/5 cells under glutamine starvation conditions (Fig. 6d). Consequently, visualization of ATAC-seq traces with integrative genomics viewer (IGV) showed that *IFRD1* deletion significantly increased chromatin accessibility of ribosome biosynthesis-related genes (Fig. 6e). Moreover, evaluation of their mRNA levels showed that these genes were comparatively upregulated in IFRD1 knockout cells following glutamine withdrawal while overexpression of H1.0 restored their levels (Fig. 6f). In general, the changes in ribosome biosynthesis-related pathways were reflected by protein synthesis alterations. Our proteomics data from the in vitro screening system (Fig. 1a; Supplementary S1) revealed that the proportion of downregulated proteins was higher than that of upregulated ones (Supplementary Fig. S13a), indicating the generally inhibited protein synthesis under glutamine starvation, which was further validated by puromycin pulse-chase assays (Supplementary Fig. S13b, c). Further, higher rates of mRNA translation in glutamine-starved IFRD1 knockout compared to WT cells could be reversed by H1.0 overexpression (Fig. 6g, h). Finally, inhibition of protein synthesis by rapamycin largely rescued cell death in IFRD1 knockout cells under glutamine starvation (Fig. 6i).

Together, these data demonstrate that the inhibition of ribosome and protein biosynthesis through the prohibition of H1.0 autophagic degradation represents a major downstream effect of IFRD1, which in turn promotes the survival of HCC cells under glutamine deprivation stress.

### IFRD1 loss enhances glutamine starvation therapy in vivo

**Fig. 7:**
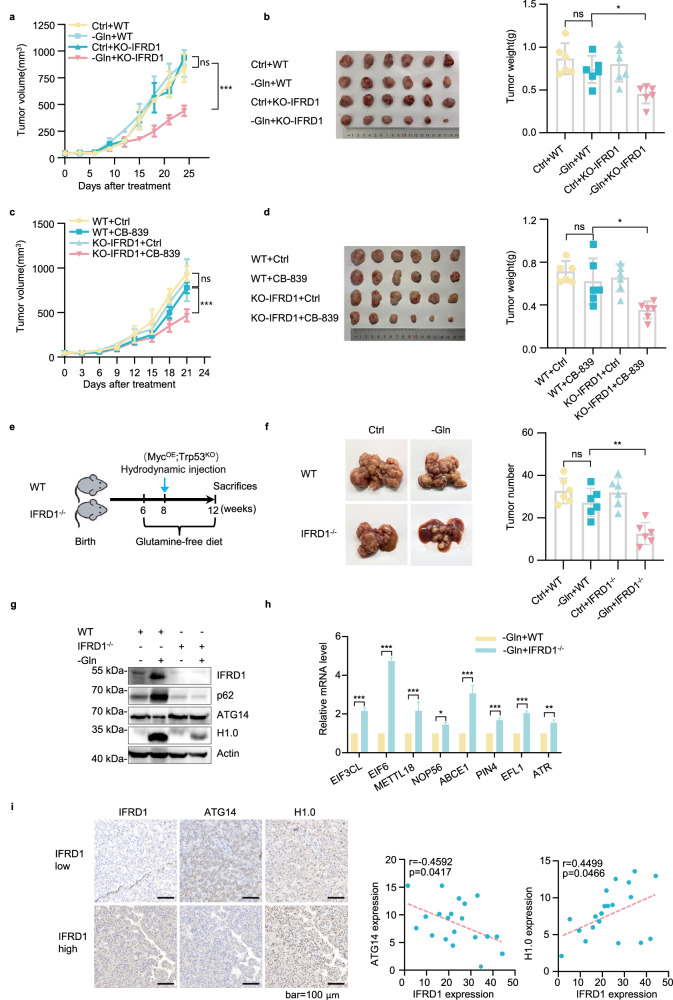
IFRD1 loss enhances glutamine starvation therapy of HCC in vivo. **a**, **b** Effect of a glutamine-free diet (–Gln, *n* = 6) compared with the matched control diet (Ctrl, *n* = 6) on subcutaneously engrafted HepG2 cells measured by tumor volume (**a**) and tumor weight (**b**). **c**, **d** Effect of CB-839 (–Gln, *n* = 6) compared with vehicle (Ctrl, *n* = 6) on subcutaneously engrafted HepG2 cells as measured by tumor volume (**c**) and tumor weight (**d**). **e** Scheme of the experimental design in WT or *IFRD1*^–/–^ mice with *Myc*^*OE*^*;Trp53*^*KO*^ autochthonous HCC. **f** Effect of a glutamine-free diet (–Gln, *n* = 6) compared with the matched control diet (Ctrl, *n* = 6) on WT or *IFRD1*^–/–^ mouse liver with *Myc*^*OE*^*;Trp53*^*KO*^ autochthonous HCC as measured by tumor number. **g** Representative immunoblots of the indicated proteins in tumors from **f**. **h** RT-qPCR analyses of ribosome biogenesis-related genes in tumors from **f**. **i** Representative immunohistochemistry images of IFRD1, ATG14, and H1.0 in 20 paired HCC tissues (left). Correlation analyses conducted between IFRD1 level and ATG14 or H1.0 level (right). Data shown are representative of three independent experiments (**g**, **h**). Data are mean ± SD; *P < 0.05; **P < 0.01; ***P < 0.001; ns, not significant by two-way ANOVA (**h**).

Lastly, we assessed the effects of IFRD1 in vivo on HCC tumorigenesis, together with potential therapeutic applications. Comparing the growth of subcutaneous HepG2 xenografts showed negligible differences between WT and IFRD1 knockout cells in mice fed a normal chow diet. However, the mice maintained on glutamine-deficient diet exhibited significantly decreased tumor volume and weights of IFRD1 knockout tumors but not WT tumors (Fig. 7a, b). Further examination of the tumor tissues using immunohistochemical staining against Ki67 showed a marked reduction in mitotic cells associated with the tumor growth retardation observed in IFRD1 knockout tumors in mice fed glutamine-deficient chow (Supplementary Fig. S14a). Moreover, consistent with our in vitro findings, the restrictive glutamine diet was sufficient to induce IFRD1 signaling as indicated by protein levels of ATG14 and H1.0 (Supplementary Fig. S14b).

These promising findings prompted us to test whether targeting IFRD1 could be exploited therapeutically in combination with clinical glutamine deprivation therapy. Towards this, we examined whether the effects of CB-839 could be amplified in cells lacking IFRD1. Indeed, comparisons of xenografted HepG2 tumors showed that CB-839 had no effect on the growth of WT tumors but significantly reduced growth of IFRD1 knockout cells (Fig. 7c, d). Thus, IFRD1 inhibition sensitizes tumors to dietary glutamine restriction.

Further, we implemented a genetic approach whereby the *IFRD1* gene was knocked out in mice and measured the effects in an autochthonous tumorigenesis model. First, CRISPR-Cas9 was used to eliminate IFRD1 expression through the targeted deletion of exon 3 (Supplementary Fig. S15a). *IFRD1*^–/–^ and WT mice were hydrodynamically injected with plasmids encoding *Myc*^*OE*^ and *Trp53*^*KO*^ to develop liver tumors^32^. Comparing homozygous WT and *IFRD1*^–/–^ mice with the *Myc*^*OE*^*;Trp53*^*KO*^ background showed that the incidence of liver tumors in WT mice was not affected by a glutamine-deficient diet, whereas the number of lesions was significantly reduced in *IFRD1*^–/–^ mice (Fig. 7e, f; Supplementary Fig. S15b). Notably, analysis of the murine liver tumors showed protein changes consistent with our data from human HCC cells (Fig. 7g). Moreover, we found that the expression levels of ribosome biosynthesis-related genes were relatively increased in glutamine-deprived *IFRD1*^–/–^ mice (Fig. 7h), indicating that IFRD1 signaling is conserved between mouse and human. Extending our analyses to clinical HCC tissues, we found a significant negative Pearson correlation between IFRD1 and ATG14 levels but a positive correlation between H1.0 and IFRD1 levels (Fig. 7i), suggesting that the IFRD1 pathway is active in clinical cases.

## Discussion

In mammals, similar to other subtypes of autophagy, nucleophagy plays a crucial role not only in cellular responses to nuclear damage and cell cycle disruption but also in cell differentiation and development, as described by Nektarios Tavernarakis^33^. In mammals, nucleophagy is primarily monitored under pathological conditions, such as neurodegeneration and cancer^34,35^. For instance, two major components of the nuclear lamina, Lamin A/C and Lamin B, have been identified as substrates of mammalian nuclear autophagy, and degradation of nuclear lamina components by autophagy has been shown to protect cells from tumorigenesis^34^. Additionally, SIRT1 has been reported to undergo LC3-mediated autophagic degradation during the aging process^35^. However, apart from these proteins, there have been few reports on novel nuclear autophagy substrates. We have discovered that under conditions of glutamine deprivation, H1.0 undergoes p62-mediated autophagy, which represents a novel member of nucleophagy substrates and the first nucleophagy substrate mediated by the autophagy receptor p62. This suggests that the nuclear autophagy pathway may have a widespread role in cellular physiology. Future research should investigate whether there are more nuclear autophagy pathway substrates and elucidate the molecular mechanisms underlying the selectivity or specificity of nuclear autophagy pathway substrate regulation in organisms.

The occurrence of autophagy can, on one hand, induce the degradation of unnecessary proteins, carbohydrates, and lipids within the cell to provide energy and re-synthesize essential proteins for cell survival, allowing cells to maintain their viability under conditions of starvation. On the other hand, sustained autophagy can lead to cell death through excessive degradation of intracellular substances. It is crucial to control this balance, which is why autophagy is often referred to as a double-edged sword. The relationship between glutamine and autophagy is even more complex. On one hand, glutamine can promote autophagy through the generation of ammonia from glutaminolysis. On the other hand, glutamine can activate the Akt/mTOR/S6k pathway and inhibit autophagy by increasing the phosphorylation of mTOR (Ser2448). We found that prolonged glutamine deficiency does not lead to excessive autophagy in cells. However, when *IFRD1* is knocked out, cells undergo autophagic exhaustion and death due to enhanced nuclear autophagy of H1.0 and overall metabolic depletion. This also explains why tumor cells do not undergo excessive autophagy during glutamine starvation therapy.

The prevailing dogma is that the ubiquitin-proteasome system (UPS) is responsible for the degradation of short-lived proteins and soluble misfolded proteins, whereas the different forms of autophagy are responsible for the elimination of long-lived proteins, insoluble proteins, whole organelles, and intracellular parasites. While these are ostensibly two separate systems, there is growing interest in their interconnections and crosstalk, which occur at multiple levels^36^. At a direct level, the targeting of autophagy components by the UPS can both positively and negatively regulate autophagy flux. For example, ubiquitination of the autophagy receptor optineurin by the E3 ligase HACE-1 enhances its association with p62 to enhance autophagy^37^, while the E3 ligase TRIM27 directs ULK1 for proteasomal degradation to dampen autophagy^38^. Notably, both exemplary studies reported their findings in the cancer context where ectopic HACE-1 expression enhances autophagy to inhibit the growth of human lung cancer xenografts, while the genetic deletion of *TRIM27* is also associated with enhanced autophagy inhibiting tumor initiation in the PyMT murine mammary tumor model but conversely promoting metastasis. Here, we found that IFRD1 regulates the recruitment of TRIM21 to ATG14, with the resulting ubiquitination and proteasomal degradation of ATG14 responsible for limiting autophagy flux under limiting glutamine conditions. Our example involving IFRD1 helps further illustrate the complex relationship between autophagy and tumorigenesis. Consistent with the idea that excessive autophagy must be restrained or otherwise cause deleterious outcomes, we found that HCC cell growth both in vitro and as xenografts benefited from IFRD1-mediated dampening of autophagy flux. Intriguingly, the same mechanism was necessary for supporting optimal rates of hepatocyte transformation in an autochthonous model but only in mice challenged with a glutamine-deficient diet. Notably, our investigations provided an unexpected downstream linkage to the autophagy-mediated degradation of H1.0 protein. The resultant control of global chromatin accessibility had major effects on limiting the expression of ribosome biosynthesis-related genes, broadly inhibiting protein synthesis under limiting glutamine conditions (Supplementary Fig. S16). MAFG was shown to be the transcription factor driving IFRD1 induction, and since MAGF is implicated in oxidative stress responses^39^, it is conceivable that IFRD1 could also influence outcomes under stress conditions other than limiting glutamine, although this postulate remains to be tested.

While our data clearly expands the knowledge base of the interaction between the UPS and autophagy, there may be more practical implications for cancer therapy. We found that the relationships between IFRD1, ATG14, and H1.0 were intact in vivo liver cancer tissues, suggesting that the signaling pathway uncovered in vitro is clinically relevant. As mentioned earlier, there is great interest in exploiting the glutamine addiction of cancer cells through drugs that target glutamine metabolism. In this regard, our study showed the potential benefits of targeting IFRD1, which markedly promotes death when cells are challenged by glutamine withdrawal, with the in vitro effects translated in vivo after placing mice on a glutamine-deficient diet. Arguably, more impressive were our findings showing the therapeutic potentiation between *IFRD1* deletion and CB-839 treatment. Neither *IFRD1* deletion nor CB-839 treatment showed antitumor activity as monotherapies, but application together resulted in significant retardation of tumor growth.

Generally, the availability of glutamine is commonly limited in tumor cells within a tumor mass due to the inadequate vasculature in the tumor microenvironment^40^. If IFRD1 expression remains consistently elevated, it contributes to enhanced tumor survival, thereby indicating a more unfavorable prognosis when there is higher expression of IFRD1. Given that IFRD1 is a stress response protein, it could be envisioned that knockdown-based targeting of IFRD1 in tumors in combination with CB-839 or similar agents may have limited off-target effects on normal cells. Nonetheless, further preclinical testing is required to confirm this point, together with additional studies to establish the relevance of IFRD1 in cancers other than HCC.

## Materials and methods

### Mouse experiments

BALB/c nude mice (female, 3–5 weeks old) were purchased from Shanghai SLAC Laboratory Animal Co., Ltd. and housed at the animal facility of the University of Science and Technology of China. After 3-day acclimatization, mice were subcutaneously injected with either WT/KO-IFRD1 HepG2 cells (5 × 10^6^ cells) or PLC/PRF/5 cells (1 × 10^7^ cells) into their right posterior flanks. When the xenografted tumors reached a volume of ~50–100 mm^3^, groups of 6 mice were randomly selected and fed either with normal chow or switched to a glutamine-free diet (Medicience Ltd.) and maintained for 4 weeks. For drug administrations, CB-839 (150 mg/kg) or vehicle was administered by oral gavage twice per day for a total of 10 doses. Tumor volumes were calculated by the modified ellipsoidal formula: tumor volume = (length × (width)^2^)/2.

Alternatively, IFRD1 knockout (*IFRD1*^–/–^) C57BL/6 mice were generated by CRISPR-Cas9-mediated deletion of exon 3 of the imprinted IFRD1 box gene under commercial arrangement with Shanghai Model Organisms Center, Inc. Following genotyping, 6-week-old backcrossed mice were separated into WT and *IFRD1*^–/–^ groups, and subjected to hydrodynamic gene delivery of plasmids with *Myc* overexpression (*Myc*^*OE*^) and knockout of *p53* (*Trp53*^*KO*^) to create an autochthonous HCC model as previously described^32^. Where indicated, a glutamine-free diet was initiated 2 weeks prior to injection and maintained for 6 weeks. All mice were humanely euthanized at experimental endpoints with tumor tissues and organs collected, imaged, and weighted before fixation or freezing as required for further analysis. All animal experiments were conducted under the approval of the Animal Management Committee of the University of Science and Technology of China (USTCCACUC212301036).

### Cell lines and culture conditions

HepG2, PLC/PRF/5, HuH7, PANC-1, SW620, SK-OV-3, HCT116, U2OS, NB4, A549 and 293T cell lines were obtained from the ATCC. Cells were routinely cultured in DMEM (Gibco) with 10% FBS (BI), 1% penicillin/streptomycin (Solarbio), and incubated at a humidified 37 °C, 5% CO_2_ atmosphere. For glutamine starvation, cells were plated in complete medium overnight, washed twice with PBS, and replenished with a glutamine-free medium for the indicated times.

### RNA-seq

Total RNA was extracted by phenol/chloroform, and RNA-seq was performed using an Illumina HiSeq 2500. Clean reads were aligned to the hg38 reference genome with Hisat2 using default parameters. Gene expression levels were counted with HT-seq using default parameters. Differential expression analysis of two groups was performed using the DESeq2 R package. DESeq2 provides statistical routines for determining differential expression in digital gene expression data using a model based on the negative binomial distribution. Genes with an adjusted *P* < 0.05 were considered to be differentially expressed. Sequencing data were deposited in the National Center for Biotechnology Information Gene Expression Omnibus database.

### Proteomics and LC-MS/MS analysis

For proteomic-seq, the indicated cells cultured with normal or glutamine-free DMEM for 24 h were lysed and subjected to trypsin digestion. Tryptic peptides were successively subjected to TMT labeling, HPLC fractionation, affinity enrichment, and data analysis. For LC-MS/MS, tryptic peptides from each sample were resolved using an EASY-nLC™ 1200 chromatographic system (Thermo Scientific) with a 300 nL/min flow rate. The resulting MS/MS data were processed using MaxQuant search engine (v1.6.1.0). Tandem mass spectra were searched against the human uniprot database (9606 entries) concatenated with the reverse decoy database. Trypsin/P was specified as a cleavage enzyme allowing up to 2 missing cleavages. The mass tolerance for precursor ions was set as 20 ppm in the first search and 4.5 ppm in the main search, and the mass tolerance for fragment ions was set as 0.04 Da. Carbamidomethyl on Cys was specified as fixed modification, and acetylation on protein N-terminal and oxidation on Met were specified as variable modifications. FDR was adjusted to < 1%.

### Cell line construction

For the generation of stable knockdown/knockout or overexpression HCC cell lines, lentiviral particles expressing shRNAs or cDNAs (see Supplementary Table S5) were prepared by transfecting 293T cells in combination with the packaging plasmids psPAX2 and pMD2.G at the ratio of 2:2:1. After 48 h, supernatants were collected, filtered with 0.45-μm filters, and used to infect cells in combination with 8 μg/mL polybrene. After 24 h, the cells were replenished with a complete medium containing puromycin. For transient overexpression, transfections were performed with the indicated plasmids (see Supplementary Table S5) using the Lipofectamine 3000 (Invitrogen) according to the manufacturer’s instructions.

### RT-qPCR

Total RNA was extracted from cells or tumors using the TRIzol reagent (Invitrogen) and reverse transcribed into cDNA by using PrimeScript^TM^ RT reagent kit (TaKaRa) according to the manufacturer’s instructions. Afterwards, RT-qPCR was implemented using the One-Step PrimeScript RT-PCR kit (TaKaRa) (primer sequences are shown in Supplementary Table S6). Relative expression values were calculated using the comparative Ct method normalized against the β-actin (for HCC cells) or 18S rRNA (for mouse liver tumor tissues) housekeeping gene.

### Western blot

Cultured cells were directly lysed in RIPA lysis buffer (Beyotime Biotechnology) containing protease inhibitors, while tumor tissues were homogenized in RIPA buffer. Lysates were centrifuged, and the supernatants were collected and subjected to BCA assays to determine protein concentrations (Beyotime Biotechnology). Equal amounts of protein were subjected to SDS-PAGE and transferred to nitrocellulose membranes. Thereafter, the membranes were first blocked with 5% defatted milk (room temperature, 30 min), and then incubated with the indicated primary antibodies (4 °C, overnight) and the corresponding species-matched horseradish peroxidase-conjugated secondary antibodies (room temperature, 1 h). Decorated bands were detected using chemiluminescence (Tanon). Antibodies are shown in Supplementary Table S7.

### Cell viability assays

Cells were first plated into 96-well plates with 2000 cells per well in a complete medium. On the following day, the medium was removed, and complete or glutamine-free medium was added. Cell viability was measured every 2 days using the Cell Counting Kit-8 (Biomiky) according to the manufacturer’s instructions. The activity of specific inhibitors was normalized by Day 5 (OD_450_) – Day 1 (OD_450_).

### Cell death assays

The indicated cells were seeded into 12-well plates overnight in complete medium. The medium was removed, and complete or glutamine-free medium was added for 48 h. Specific inhibitors were added at the indicated times, and cells were subjected to PI and Hoechst staining. The percentage of cell death was normalized by PI-positive cells/Hoechst-staining cells.

### Dual-luciferase reporter and mammalian two-hybrid assays

Dual-luciferase reporter assays were performed according to the manufacturer’s instructions (Promega). For the mammalian two-hybrid assay, complementary DNAs for *TRIM21* and *ATG14* were cloned into the pACT and pBIND vectors, respectively, and these constructs were co-transfected with the pG5luc luciferase vector into target cells according to the manufacturer’s instructions (Promega).

### ChIP assays

ChIP assays were performed with the indicated antibodies and primers (see Supplementary Tables S6, S7) using the kit purchased from Beyotime Biotechnology according to the manufacturer’s instructions.

### Fluorescence and histochemical immunostaining

Cells were first adhered to glass coverslips and subjected to glutamine starvation for 48 h as required. For immunostaining against IFRD1, H3K9me3, and Lamin A/C, the cells were rinsed with PBS and fixed with 4% formaldehyde for 10 min at 37 °C, followed by permeabilization in 0.2% Triton X-100 for 3 min. Alternatively, for the detection of LC3B, RFP-GFP-LC3B, Ptdlns3P, ZFYVE1-GFP, P62, and H1.0, the cells were rinsed with PBS and fixed in 100% methanol for 15 min at −20 °C. Thereafter, the coverslips were rinsed in PBST (0.05% Tween-20 in PBS) three times for 5 min, blocked in 1% BSA at room temperature for 30 min, and then incubated with primary antibody overnight at 4 °C. On the next day, after being washed three times with PBST, the coverslips were incubated with appropriate fluorescence-labeled secondary antibodies at room temperature for 30 min. Finally, after further washing, the coverslips were mounted in an antifade mounting medium containing DAPI, and cell images were captured using a Zeiss LSM880 Airyscan. Alternatively, immunohistochemical staining of tumor sections was conducted by Wuhan Servicebio Technology Co., Ltd.

### Transmission electron microscopy

Glutamine-starved cells (~1 × 10^7^) were washed with cold PBS, prefixed in 4% formaldehyde solution for 2 min, gently pelleted by centrifugation at low speed, fixed in 2.5% glutaraldehyde at 4 °C for 12 h, and postfixed in 1% OsO4 for 1 h at room temperature. The specimens were then dehydrated via graded ethanol solutions, saturated in a propylene oxide/epoxy resin mix (1:1) for 1 h, and then embedded in pure epoxy resin for 2 h. The embedded specimens were then baked at 45 °C for 12 h and at 72 °C for 24 h, and ultrathin sections (70 nm) were cut using a Leica UC-7 ultramicrotome with sections collected on copper grids. After the sections were stained with lead citrate, the specimens were imaged using a transmission electron microscope.

### Subcellular fractionation

For cytosol/nucleus extraction, the indicated cell suspensions were resuspended with cold hypotonic buffer (10 mM HEPES, pH 7.9, 10 mM KCl, 1.5 mM MgCl_2_, 0.5 mM β-mecaptoethanol) containing protease inhibitor cocktail on ice for 20 min, and 10% NP-40 was added for another 5 min to a final concentration of 1% NP-40. The homogenate was centrifuged at 1000 rpm for 15 min at 4 °C, and the supernatant fraction enriched in cytosolic components was collected and stored. The pellet was rinsed twice with cold PBS and centrifuged at 1000 rpm for 5 min at 4 °C to collect the pellet as nuclear fraction. Alternatively, ER fractions were extracted using the kit from Beijing BioRab Technology Co., Ltd., according to the manufacturer’s instructions.

### Immunoprecipitation

The indicated cells were lysed with IP lysis buffer (50 mM Tris-HCl, pH 7.4, 150 mM NaCl, 1 mM EDTA, 5% glycerol, 0.4% Triton X-100, 0.4% NP-40) supplemented with a protease inhibitor cocktail, and cell lysates were clarified by centrifugation at 4 °C for 30 min. The supernatants were incubated with anti-FLAG M2 beads or other specific antibodies pre-coupled to protein A + G agarose beads at 4 °C for 4 h. The beads were washed with IP wash buffer (50 mM Tris-HCl, pH 7.4, 150 mM NaCl, 1 mM EDTA, and protease inhibitor cocktail). The immunoprecipitated complexes were eluted in SDS-PAGE loading buffer and subjected to western blot analysis.

### In vitro protein–protein binding assays

*Escherichia coli* cells (BL21) were transformed with the indicated GST/GST-fusion plasmids or His-fusion plasmids and lysed with either GST lysis buffer (PBS, 0.5% Triton X-100 and protease inhibitor cocktail) or His lysis buffer (50 mM NaHPO_4_, 300 mM NaCl, 10 mM imidazole, 5% glycerol, 0.5% Triton X-100 and protease inhibitor cocktail) by sonication, respectively. After centrifugation, supernatants were incubated with glutathione-coupled resin (Beyotime Biotechnology) or Ni resins (Beyotime Biotechnology) at 4 °C for 4 h followed by washing with the respective GST wash buffer (PBS, 0.1% Triton X-100) or His wash buffer (50 mM NaHPO_4_, 300 mM NaCl, 10 mM imidazole, 0.1% Triton X-100 and protease inhibitor cocktail). The His-fused proteins were eluted using His elution buffer (50 mM NaHPO_4_, 300 mM NaCl, 200 mM imidazole, 0.1% Triton X-100, and protease inhibitor cocktail).

To investigate the binding, the indicated soluble His-fused proteins were mixed with immobilized GST-fusion protein on the binding resin and incubated at 4 °C for 3 h with rotation. Afterwards, the samples were analyzed by western blotting.

### ATAC-seq

ATAC-seq libraries were generated following the manufacturer’s protocol (NOVOPROTEIN, N248) with library quality control and sequencing performed by Genewiz. The data analysis workflow first involved adapter trimming and removal of low-quality reads using Trim_Galore software in paired-end mode. Clean reads were then mapped to the hg38 reference genome with Bowtie2 followed by removing PCR duplicates using Picard. The genome browser tracks in bigwig format were produced from merged replicates using samtools and peaks were called using MACS2 using parameters “-f BAMPE --nomodel --shift -100 --extsize 200 –nomodel”. The bigwig data were visualized using the IGV software (Broad Institute). Peak visualizations were prepared using Deeptools. The sequencing data were deposited in the National Center for Biotechnology Information Gene Expression Omnibus database.

### Protein synthesis assay

Protein synthesis was assessed as described^41^. Briefly, cultured cells were pulsed with 10 μg/mL puromycin for 30 min, and chased in puromycin-free media for 1 h. The cell lysates were subjected to western blot analysis using anti-puromycin antibodies (see Supplementary Table S7).

### Colony formation assays

Cells were seeded into 6-well plates with 1000 cells per well overnight, and the medium was replaced with fresh complete medium or glutamine-free medium every 2 days. After 7 days, colonies were fixed with 4% formaldehyde (room temperature, 10 min) followed by staining with crystal violet staining solution (room temperature, 20 min) and washing with ddH_2_O. Stained colonies were allowed to dry overnight, and the wells were imaged.

### Human subjects

Tumor tissue microarray containing 20 cases of liver cancer were purchased from Shanghai Outdo Biotech Co., Ltd. (HLivHCC050PG01). Specimens were obtained retrospectively as paraffin sections, with no impact on the patient’s clinical diagnosis and treatment, nor the study posing any physical or mental burden. The study was compliant with all relevant ethical regulations, including the protection of patient personal information, and no patient specimens or information was used when they explicitly refused to participate in a research study. The study was conducted in adherence to the Declaration of Helsinki and approved by the Animal Management Committee of the University of Science and Technology of China (2023KY191).

### Analysis of the relationship between the expression levels of IFRD1 and glutamine metabolism-related genes

Existing, publicly available scRNA-seq data sets were used in this study to evaluate the relationship between the expression levels of IFRD1 and glutamine metabolism-related genes, including the data by Sun et al.^23^ from China National GeneBank Database (CNP0000650), and our group data^22^ published previously.

For scRNA-seq data, we downloaded processed data from the China National GeneBank DataBase (CNGBdb, http://ftp.cngb.org/pub/CNSA/data3/CNP0000650/Single_Cell/CSE0000008/). After extracting tumor cell subpopulations, we used the Seurat^42^ package for normalization, dimensionality reduction, and visualization. Regarding the spatial transcriptomics data, the processing methodology has been previously described^22^.

The gene sets related to glutamine metabolism was downloaded from the Molecular Signatures Database (MSigDB, https://www.gsea-msigdb.org/gsea/msigdb/index.jsp), using the gene set named as “GOBP_GLUTAMINE_FAMILY_AMINO_ACID_METABOLIC_PROCESS”. The Seurat package was employed to evaluate the levels of glutamine metabolism-related genes using the “Add Module Score” function, followed by visualization using “Feature Plot” and “Spatial Feature Plot” function.

### Statistics analysis

All results were obtained from three independent replications. Statistical tests were accomplished by GraphPad Prism 8.0.2 using Student’s two-tailed unpaired *t*-test for pairwise comparisons, one-way ANOVA for multiple comparisons, two-way ANOVA for multiple comparisons involving two independent variables, or by log-rank test for comparisons of survival distributions of two groups. Data are presented as mean ± SD of at least three independent experiments. A *P* value < 0.05 was considered statistically significant.

### Supplementary information

Supplementary Figures 1–16

Supplementary Table S1

Supplementary Table S2

Supplementary Table S3

Supplementary Table S4

Supplementary Table S5

Supplementary Table S6

Supplementary Table S7

## Data Availability

The RNA-seq and ATAC-seq raw data in this study have been deposited to the NCBI with the accession number GSE217406.
