# Supplementary material for: IFRD1 promotes tumor cells “low-cost” survival under glutamine starvation via inhibiting histone H1.0 nucleophagy
Source: Cell Discov. 2024 May 28;10:57. doi: 10.1038/s41421-024-00668-x (PMC11130292; doi:10.1038/s41421-024-00668-x)
Supplement: Supplementary file 1 — Supplementary Figures 1–16 [file 41421_2024_668_MOESM1_ESM.pdf]

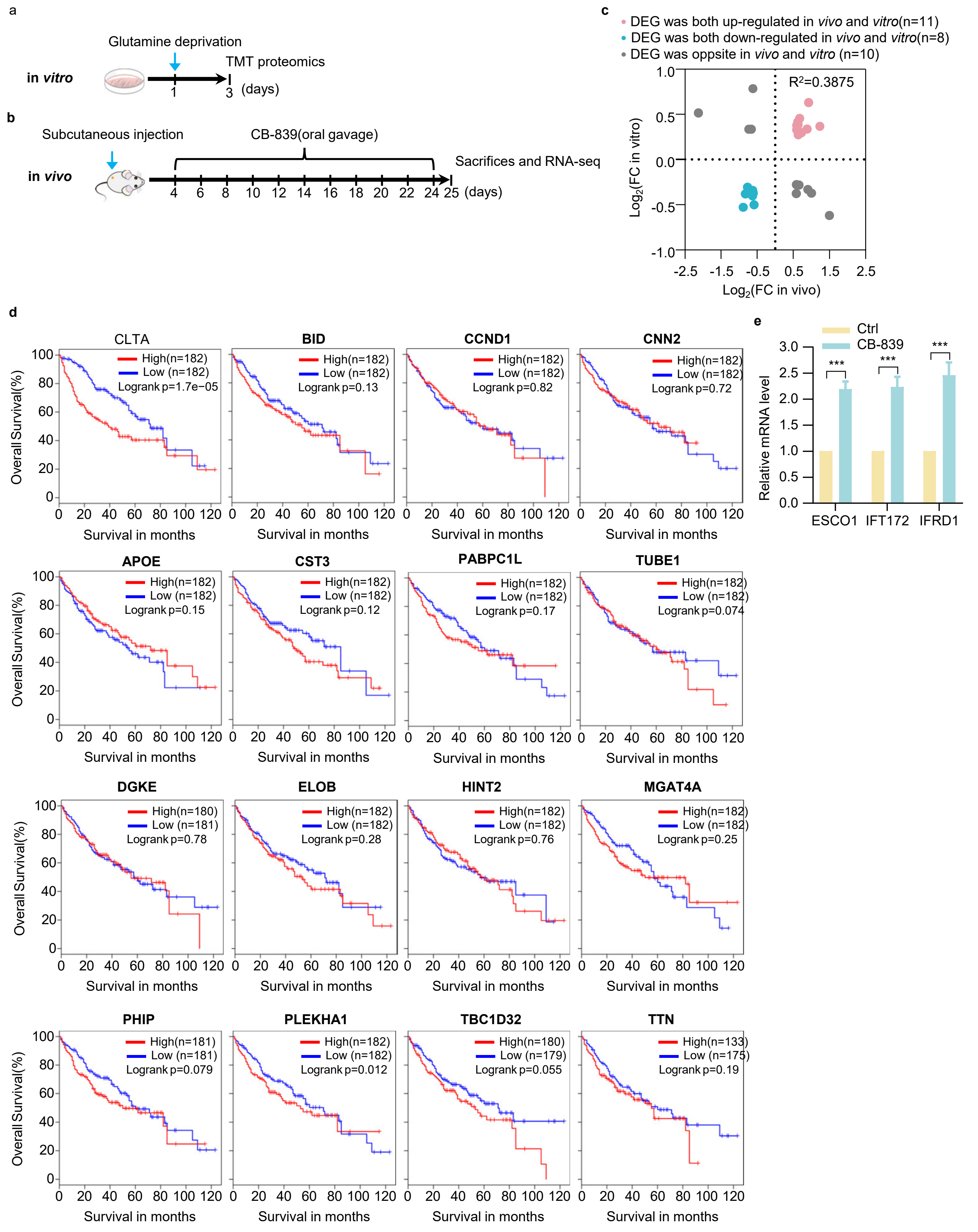

Fig S1. Selection of key regulators on HCC cells adapt to glutamine starvation.
a. Schematic of detailed experimental scheme in vitro.
b. Schematic of detailed experimental scheme in vivo.
c. Correlation analyses conducted between differentially expressed genes (DEG) in protein and mRNA expression for 29 common hits in vivo and in vitro screening systems. R^2, Pearson correlation coefficients.
d. Kaplan-Meier survival curves of overall survival in liver cancer patients from the TCGA based on 16 genes expression.
e. RT-qPCR analyses of ESCO1, IFT172 and IFRD1 in nude mouse bearing PLC/RFP/5 liver cancer tumor treated with vehicle and CB-839 respectively.
(e) represent three independent experiments, (e) Data are mean ± SD, ***P < 0.001 by two-tailed unpaired Student's t-test.

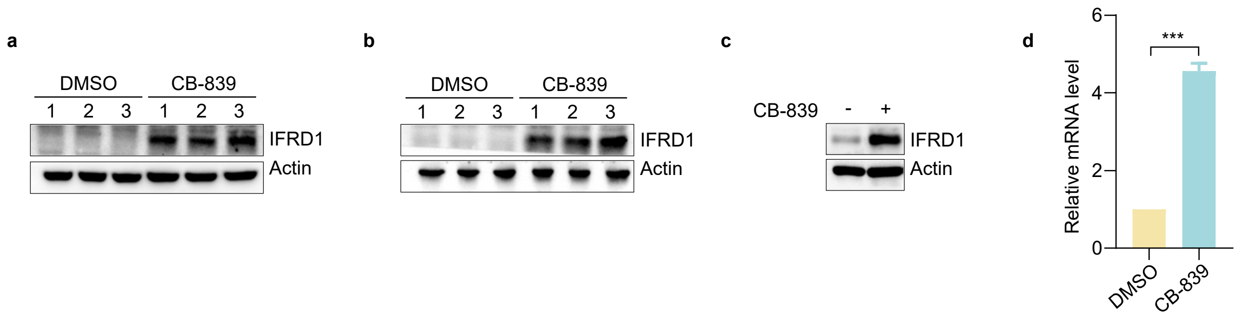

Fig S2. CB-839 treatment induces IFRD1 expression both in vivo and vitro.
a. Western blot analysis of expression of IFRD1 in nude mouse bearing HepG2 liver cancer tumor treated with vehicle and CB-839 respectively.
b. Western blot analysis of expression of IFRD1 in nude mouse bearing PLC/RFP/5 liver cancer tumor treated with vehicle and CB-839 respectively.
c. Western blot analysis of expression of IFRD1 in PLC/RFP/5 cell treated with DMSO and CB-839 (5 μm) respectively for 24h.
d. RT-qPCR analyses of IFRD1 in PLC/RFP/5 cell treated with DMSO and CB-839 (5 μm) respectively for 24h.
(a-d) represent three independent experiments, (d) data are mean ± SD, **P < 0.01; ***P < 0.001 by two-tailed unpaired Student's t-test.

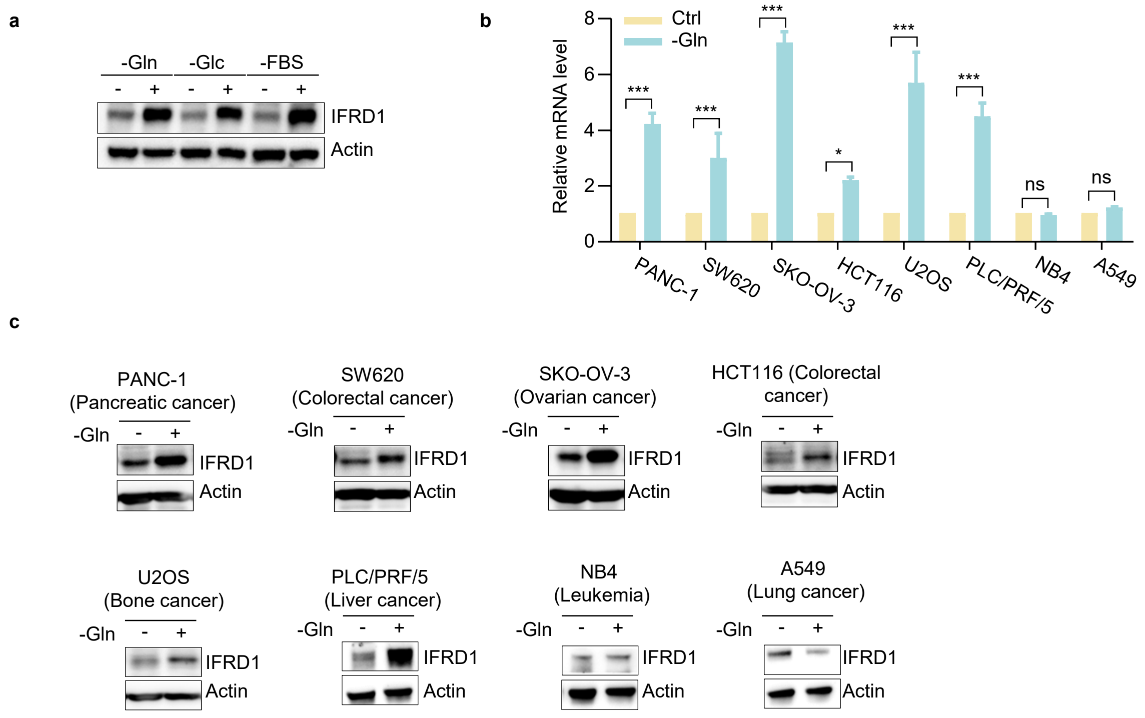

Fig S3. IFRD1 is induced in glutamine-starved cancer cells.

a. Western blot analysis of expression of IFRD1 in HepG2 cultured under normal, glutamine starvation, glucose starvation (-Glc) and serum starvation (-FBS) conditions for 48 h.

b. RT-qPCR analyses of IFRD1 in various cancer cells cultured under normal or glutamine starvation conditions for 48 h.

c. Western blot analysis of expression of IFRD1 in various cancer cells cultured with normal or glutamine starvation medium for 48 h.

(a-c) represent three independent experiments, (b) data are mean ± SD, **P < 0.01; ***P < 0.001 by two-tailed unpaired Student's t-test.

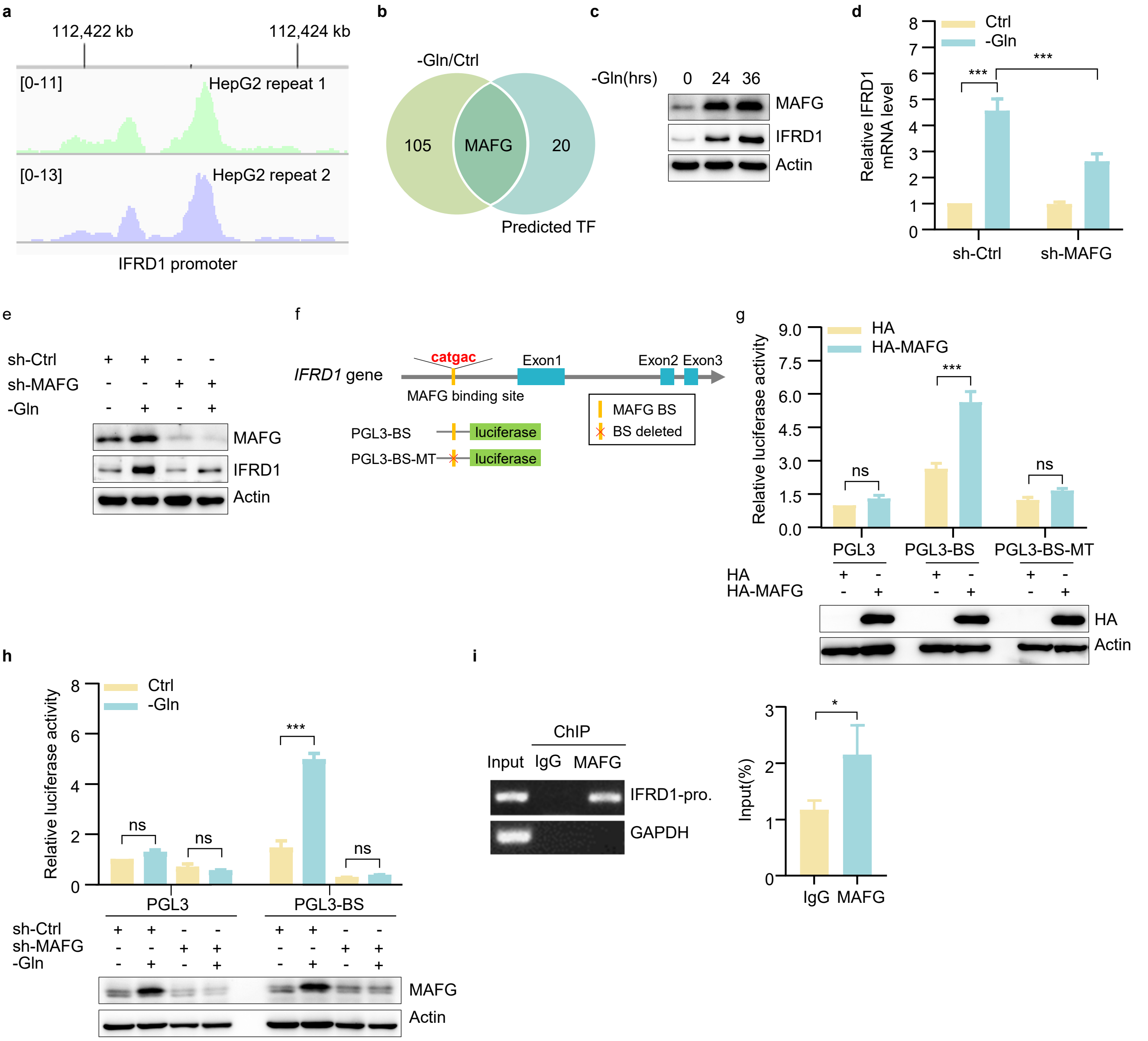

Fig S4. Transcription factor MAFG induces IFRD1 expression under glutamine starvation.

a. Genome browser traces of ATAC-seq (ENCAN074ZCE) peaks at IFRD1 promoter.

b. Overlap of predicated transcription factors based on ATAC-seq peaks detected on IFRD1 promoter and glutamine-starved versus normal HepG2 proteome data sets (FC ≥ 1.5, p < 0.05). The full list of regulators is provided in Supplementary Table S2.

c. Western blot analysis of expression of MAFG and IFRD1 in HepG2 cells after cultured under normal or glutamine starvation conditions for the indicated hours.

d. RT-qPCR analyses of IFRD1 in sh-Ctrl or sh-MAFG HepG2 cells cultured with normal or glutamine starvation medium for 48 h.

e. Western blot analysis of expression of IFRD1 in sh-Ctrl or sh-MAFG HepG2 cells cultured with normal or glutamine starvation medium for 48 h.

f. Schematic of predicted MAFG binding sites on the promoter region of IFRD1. The putative IFRD1 promoter binding sites (BS) and mutation (BS-mutation) was cloned into the PGL3.

g. Dual luciferase assays determined the transcriptional activity of BS or BS-MT sites in HepG2 cells transfected with HA-control or HA-MAFG.

h. Dual luciferase assays determined the transcriptional activity of BS or BS-MT sites in sh-Ctrl or sh-MAFG HepG2 cells cultured under normal or glutamine starvation conditions.

i. Chromatin immunoprecipitation (ChIP) assays performed in HepG2 cells against negative control IgG and anti-MAFG antibodies, semiquantitative PCR was performed using ChIP primers indicated in Supplementary Table S6. Enrichment of IFRD1 promoter (IFRD1-pro.) were imaged in left panel and quantified in right panel. GAPDH as a negative control.

(c-e, g-i) represent three independent experiments, (d, g-i) data are mean ± SD, *P < 0.05; ***P < 0.001; ns, not significant by two-tailed unpaired Student's t-test or two-way ANOVA.

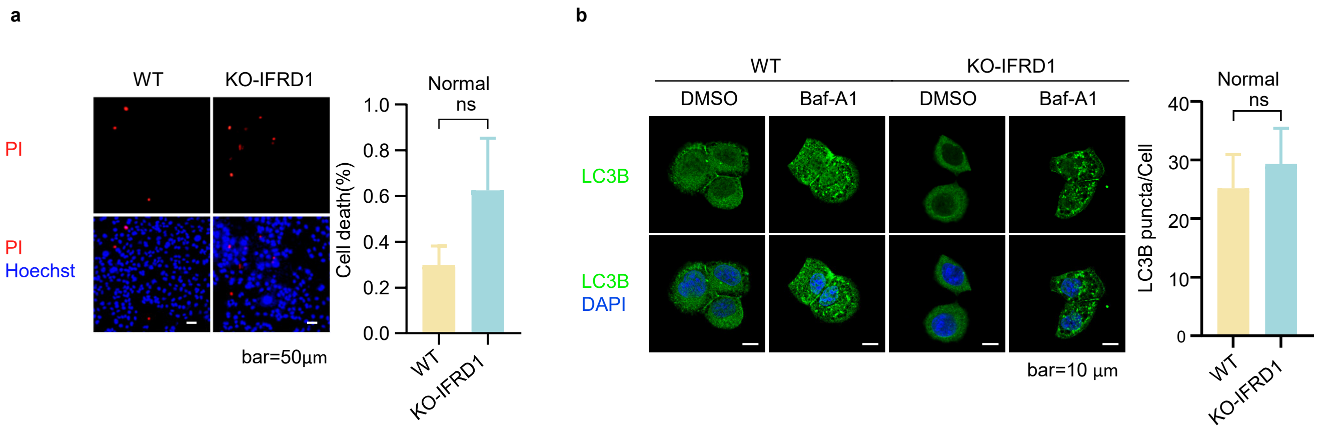

Fig S5. IFRD1 deletion doesn't affect autophagy flux and cell death under normal conditions.

a. Fluorescence microscopy images of WT and KO-IFRD1 PLC/PRF/5 cells cultured under conditions and followed by double staining with PI and Hoechst 33342 (Left). PI and Hoechst 33342 staining cells were counted and the percentage of PI-positive cells were then quantified. Scale bar, 50 μm.

b. Representative images of LC3B immunostaining in WT and KO-IFRD1 PLC/PRF/5 cells under conditions with DMSO or Baf-A1 (400 nM) for 2 h (Left). DAPI were indicated as nuclear. Percentage of LC3B puncta per cell in WT and KO-IFRD1 PLC/PRF/5 cells with Baf-A1 were quantified (n=2) (Right). Scale bars, 20 μm.

(a-b) represent three independent experiments, (a-b) data are mean ± SD; ns, not significant by two-tailed unpaired Student's t-test.

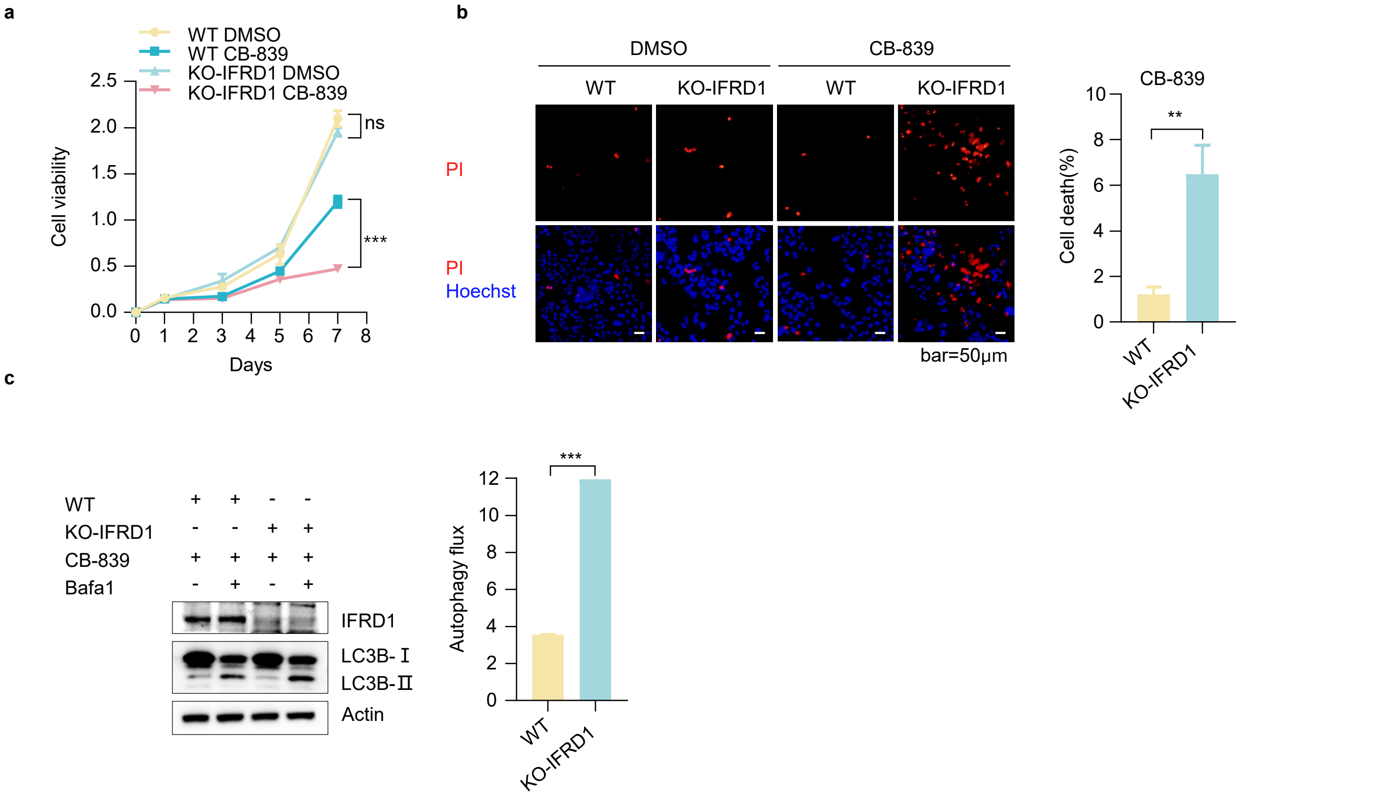

Fig S6. IFRD1 deletion enhances autophagy flux and promotes cell death under treatment of CB-839.

a. Cell viability of WT and KO-IFRD1 PLC/PRF/5 cells under DMSO or CB-839 treatment.

b. Fluorescence microscopy images of WT and KO-IFRD1 PLC/PRF/5 cells treated with CB-839 for 48 h and followed by double staining with PI and Hoechst 33342 (Left). PI and Hoechst 33342 staining cells were counted and the percentage of PI-positive cells were then quantified. Scale bar, 50 μm.

c. Representative images of LC3B and Actin (loading control) immunoblots of WT and KO-IFRD1 PLC/PRF/5 cells under CB-839 treatment for 24 h with or without Baf-A1 (400 nM) for the last 2 h (Left). Ratio of (LC3B-II + Baf-A1 /Actin)/ (LC3B-II - Baf-A1 /Actin) were indicated as autophagy flux (Right).

(a-c) represent three independent experiments, (a-c) data are mean ± SD; **$P < 0.01$; ***$P < 0.001$; ns, not significant by two-tailed unpaired Student's t-test.

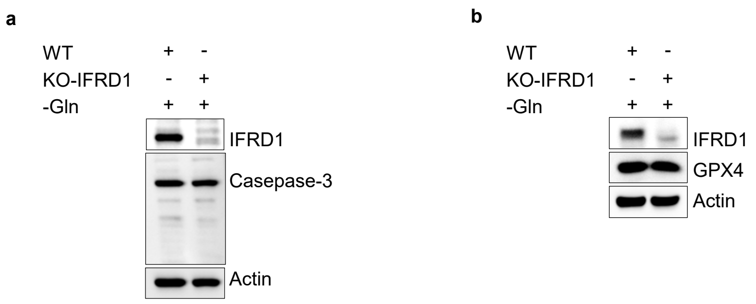

Fig S7. IFRD1 deletion-induced cell death is independent of apoptosis and ferroptosis.
a-b. Representative images of indicated proteins immunoblots of WT and KO-IFRD1 PLC/PRF/5 cells under glutamine starvation conditions for 48 h. (a-b) represent three independent experiments.

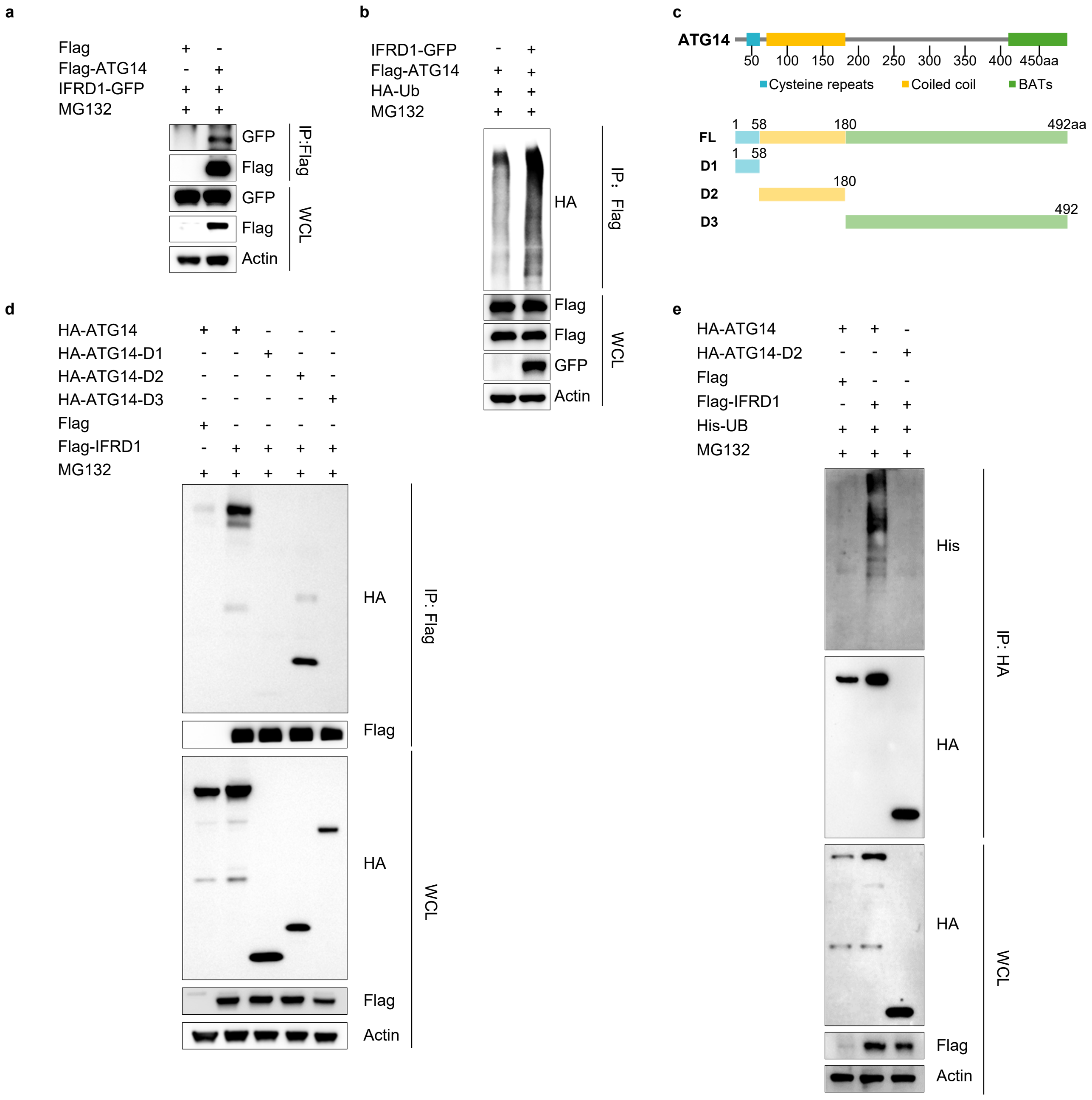

Fig S8. IFRD1 promotes ubiquitination of ATG14.
a. Co-IP analysis of the interaction between IFRD1 and ATG14 in HepG2 cells.
b. Ubiquitination level of ATG14 in the presence or absence of IFRD1 in HepG2 cells.
c. Schematic diagram of domain regions of ATG14.
d. Co-IP analysis of the interaction between IFRD1 and ATG14 domain in HepG2 cells.
e. Ubiquitination level of ATG14 domain in the presence of IFRD1 in HepG2 cells.
(a-b, d-e) represent three independent experiments.

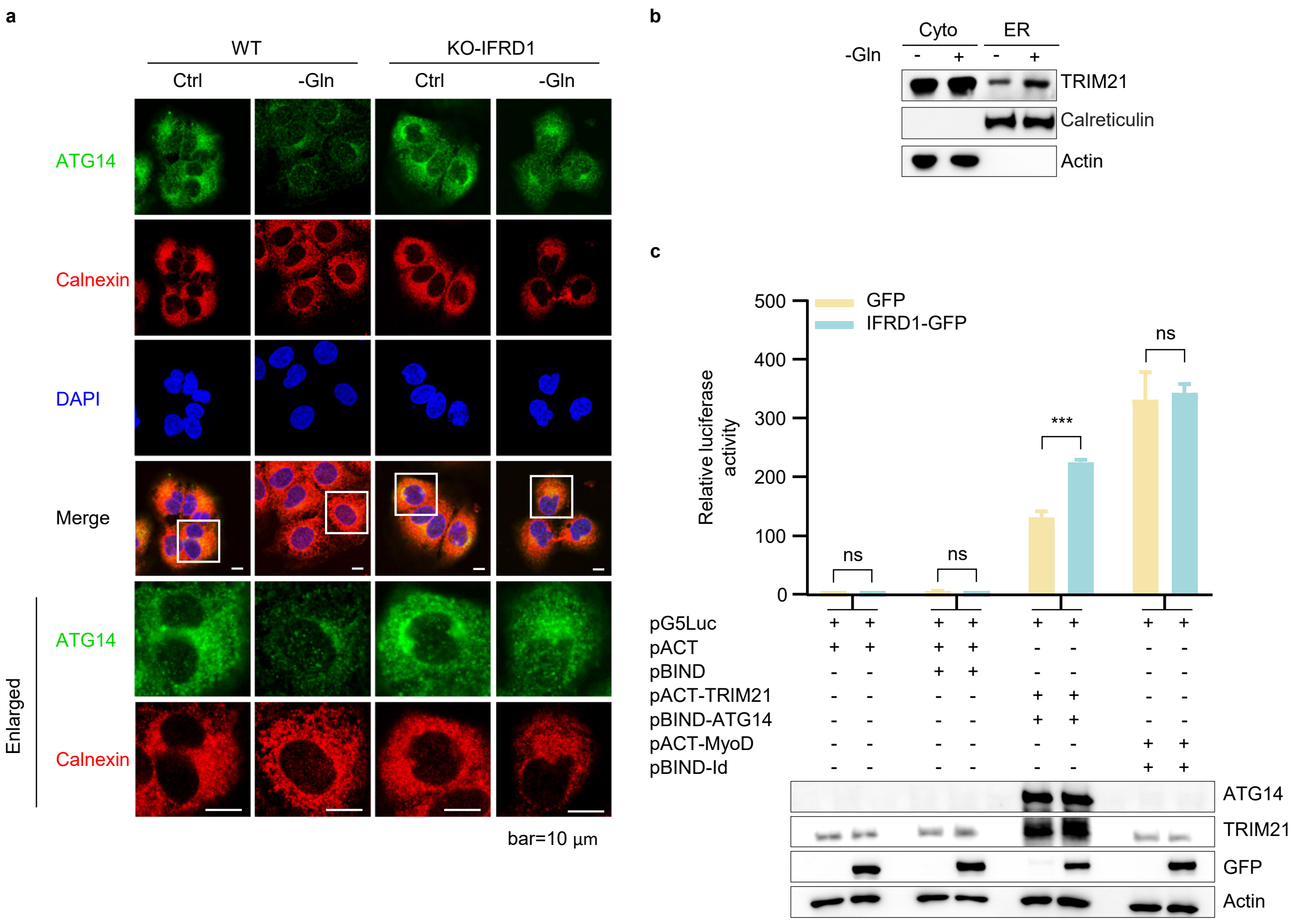

Fig S9. **IFRD1 facilitates TRIM21-ATG14 interaction on endoplasmic reticulum.**

a. Representative confocal images of ATG14 in WT and KO-IFRD1 PLC/PRF/5 cultured with normal or glutamine starvation medium for 48 h. Calnexin was indicated as ER, DAPI was indicated as nuclear. Insets: magnified views of the regions in the white boxes. Scale bar, 10 μm.

b. Representative images of immunoblots of TRIM21 in the cytoplasm (Cyto) and endoplasmic reticulum (ER)-enriched fractions extracted from normal or glutamine-starved HepG2 cells. Calreticulin was indicated as ER, Actin was indicated as cytoplasm.

c. Mammalian two-hybrid assays measuring interactions between ATG14 and TRIM21 in the presence and absence of IFRD1.

(a-c) represent three independent experiments, (c) data are mean ± SD; ***P < 0.001; ns, not significant by two-tailed unpaired Student's t-test.

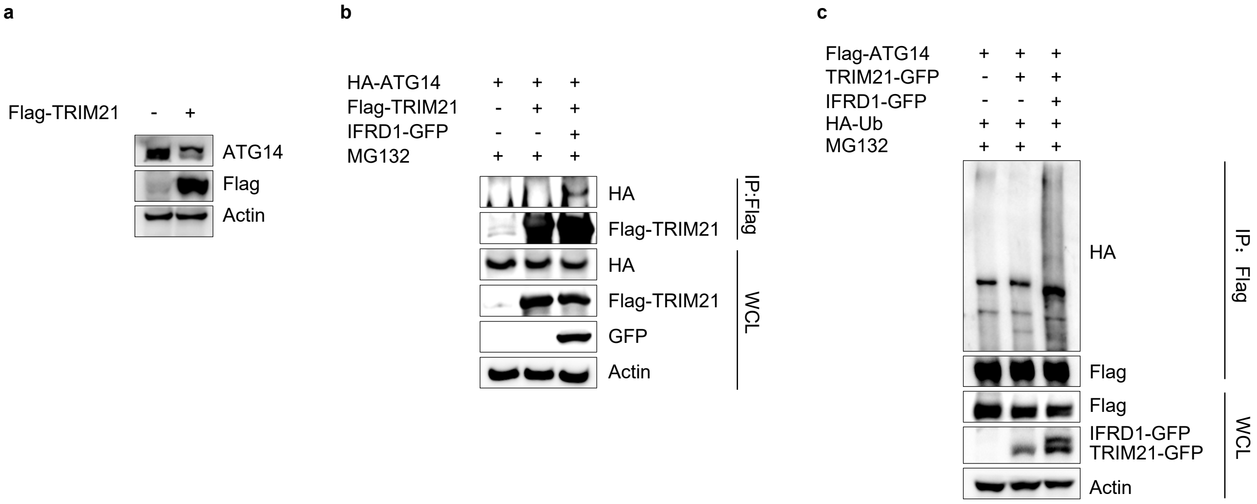

Fig S10. IFRD1 promotes TRIM21-mediated ubiquitination of ATG14.
a. Representative images of immunoblots of ATG14 in HepG2 cells expressing Flag or Flag-TRIM21.
b. IP analyses of the interaction of TRIM21 and ATG14 in the presence and absence of IFRD1.
C. IP analyses of TRIM21-mediated ubiquitination level of ATG14 in HepG2 cells in the presence and absence of IFRD1.
(a-c) represent three independent experiments.

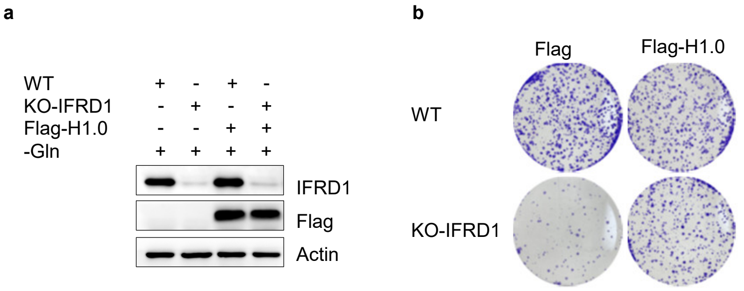

Fig S11. IFRD1 promotes growth of HCC cells by stabilizing H1.0.

a. Representative images of immunoblots of IFRD1 and Flag-H1.0 in WT and KO-IFRD1 PLC/PRF/5 cells expressing Flag or Flag-H1.0 cells cultured with normal or glutamine starvation medium for 36 h.

b. Clonogenic assays of WT and KO-IFRD1 PLC/PRF/5 cells expressing Flag or Flag-H1.0 cultured under glutamine starvation conditions for 7 days.

(a, b) represent three independent experiments.

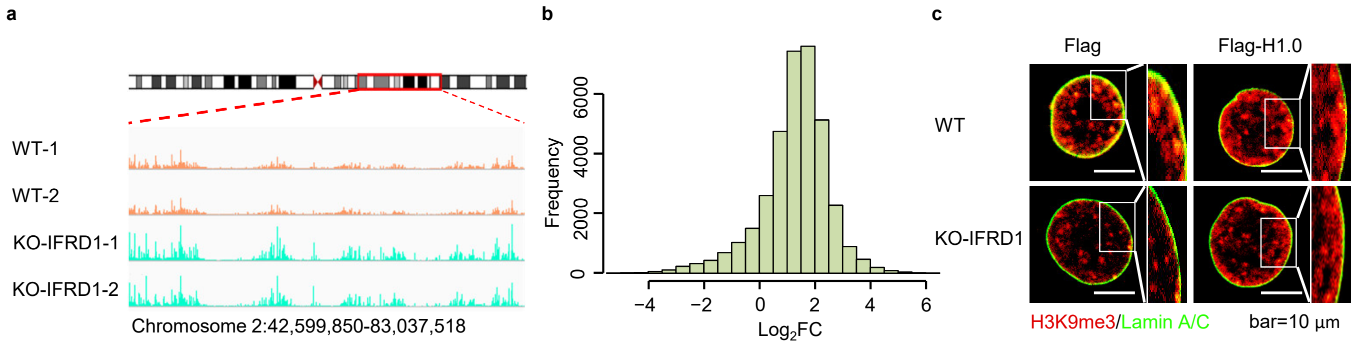

Fig S12. IFRD1 deletion enhances chromatin accessibility and heterochromatin formation under glutamine starvation.

a. Track views of chromatin accessibility based on ATAC sequencing across the chromosome 2:42,599,850-83,037,518 region in WT and KO-IFRD1 PLC/PRF/5 cells cultured with normal or glutamine starvation medium for 36 h.

b. Frequency distribution of log2FC based on chromatin accessibility peaks between WT and KO-IFRD1 PLC/PRF/5 cells cultured with normal or glutamine starvation medium for 36 h.

c. Representative images H3K9me3 and Lamin A/C immunostaining in WT and KO-IFRD1 PLC/PRF/5 cells expressing Flag or Flag-H1.0 cultured under glutamine starvation conditions for 36 h.

(c) represent three independent experiments.

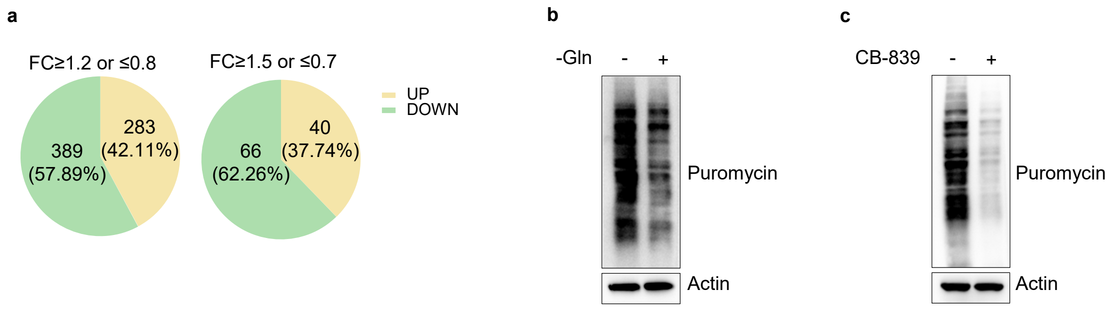

Fig S13. Glutamine starvation inhibits general protein synthesis.

a. Differentially expressed proteins (FC ≥ 1.2 or ≤ 0.8, P<0.05; FC ≥ 1.5 or ≤ 0.7, P<0.05) from in vitro screening system assigned to up and down groups and their percentage of the total are indicated.

b. Protein synthesis (assessed by puromycin pulse-chase) in PLC/PRF/5 cultured under normal or glutamine starvation conditions for 48 h.

c. Protein synthesis (assessed by puromycin pulse-chase) in PLC/PRF/5 treated with DMSO or CB-839 (5μm) respectively for 24 h.

(b-c) represent three independent experiments.

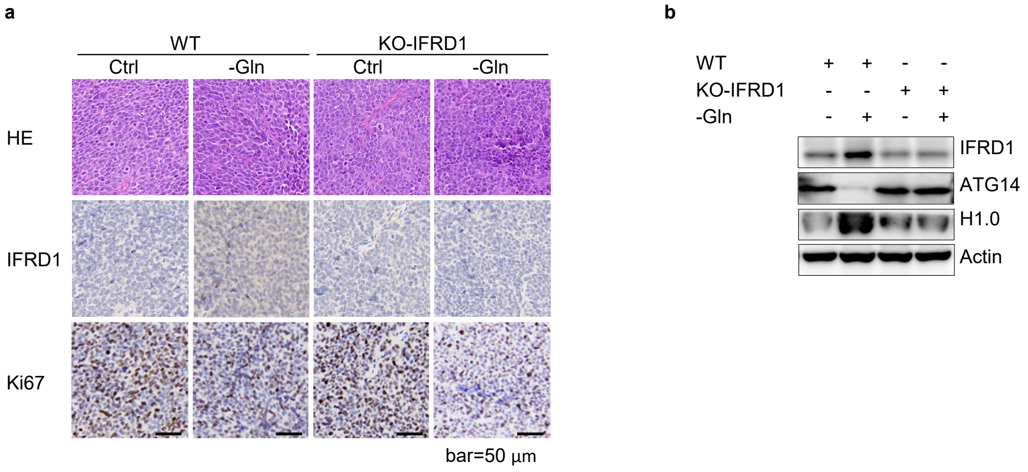

Fig S14. IFRD1 promotes tumor growth and participates in IFRD1-ATG14-H1.0 signal pathway in vivo.
a. Representative IHC images Immunohistochemistry of IFRD1 and Ki67 of tumors in Fig 7b.
b. Representative images of immunoblots of indicated proteins in Fig 7b tumors.
(a-b) represent three independent experiments.

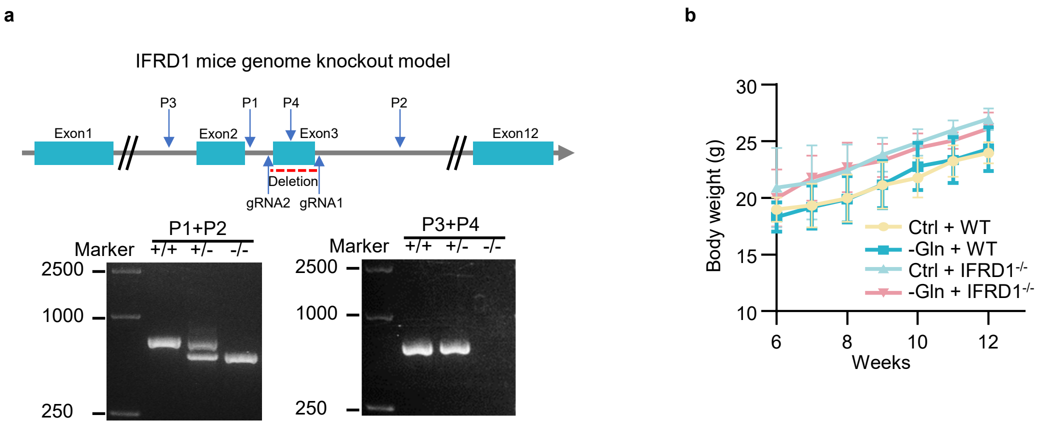

Fig S15. Construction of IFRD1$^{-/-}$ autochthonous HCC mice modes.

a. Illustration of the CRISPR-Cas9-based IFRD1 gene-knockout strategy in mice showing the location of the CRISPR guide sequences along with the PCR primers used for the genotyping assays (upper). Dual PCR reactions employing Forward (F)–Reverse (R) primers and the Deletion (Del)–Reverse primers enable validation of wildtype (+/+), heterozygous (+/−), and KO-IFRD1 (−/−) mice (lower).

b. Body weight measurements of mice in Fig 7e.

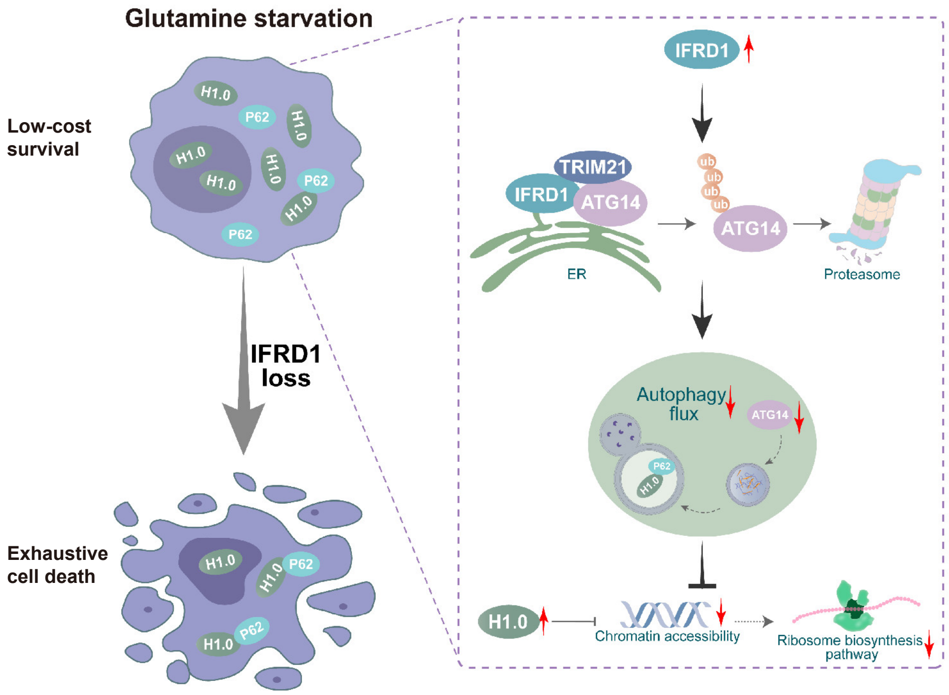

Fig S16. The mode of IFRD1 in protecting cancer cells from exhaustive death induced by glutamine starvation.
IFRD1 is induced under glutamine starvation to inhibit autophagy by promoting the proteasomal degradation of the key autophagy regulator ATG14 in a TRIM21-dependent manner. The resulting stabilized histone H1.0 serves to restrain global chromatin accessibility. Conversely, targeting IFRD1 in the glutamine-deprived state increases autophagy flux, triggering cancer cell exhaustion death. This effect is largely realized through the nucleophagic degradation of histone H1.0 and the ensuing unchecked increases in ribosome and protein biosynthesis associated with globally enhanced chromatin accessibility.
